# *Ephedra foeminea* as a Novel Source of Antimicrobial and Anti-Biofilm Compounds to Fight Multidrug Resistance Phenotype

**DOI:** 10.3390/ijms24043284

**Published:** 2023-02-07

**Authors:** Shurooq Ismail, Rosa Gaglione, Marco Masi, Srichandan Padhi, Amit K. Rai, Ghadeer Omar, Alessio Cimmino, Angela Arciello

**Affiliations:** 1 Department of Chemical Sciences, University of Naples Federico II, Via Cintia 21, I-80126 Naples, Italy; 2 Department of Biology and Biotechnology, An-Najah National University, Nablus 97300, Palestine; 3 Istituto Nazionale di Biostrutture e Biosistemi (INBB), 00136 Rome, Italy; 4 Institute of Bioresources and Sustainable Development, Imphal, Manipur 795004, India

**Keywords:** multidrug resistant infections, plant-derived drugs, bioguided purification, antimicrobial monoterpenoid phenols, antimicrobial glycosylated flavonoids

## Abstract

Plants are considered a wealthy resource of novel natural drugs effective in the treatment of multidrug-resistant infections. Here, a bioguided purification of *Ephedra foeminea* extracts was performed to identify bioactive compounds. The determination of antimicrobial properties was achieved by broth microdilution assays to evaluate minimal inhibitory concentration (MIC) values and by crystal violet staining and confocal laser scanning microscopy analyses (CLSM) to investigate the antibiofilm capacity of the isolated compounds. Assays were performed on a panel of three gram-positive and three gram-negative bacterial strains. Six compounds were isolated from *E. foeminea* extracts for the first time. They were identified by nuclear magnetic resonance (NMR) spectroscopy and mass spectrometry (MS) analyses as the well-known monoterpenoid phenols carvacrol and thymol and as four acylated kaempferol glycosides. Among them, the compound kaempferol-3-*O*-α-L-(2″,4″-di-*E*-*p*-coumaroyl)-rhamnopyranoside was found to be endowed with strong antibacterial properties and significant antibiofilm activity against *S. aureus* bacterial strains. Moreover, molecular docking studies on this compound suggested that the antibacterial activity of the tested ligand against *S. aureus* strains might be correlated to the inhibition of Sortase A and/or of tyrosyl tRNA synthase. Collectively, the results achieved open interesting perspectives to kaempferol-3-*O*-α-L-(2″,4″-di-*E*-*p*-coumaroyl)-rhamnopyranoside applicability in different fields, such as biomedical applications and biotechnological purposes such as food preservation and active packaging.

## 1. Introduction

Humans have always used plants as a natural source of food, animal feed and medical options to treat several diseases [1]. Indeed, conventional medicinal plants have been reported to contain compounds endowed with antimicrobial, antifungal, anti-inflammatory, antiviral and anticancer properties [2]. Among compounds responsible for these interesting and promising bioactivities, an important role is played by essential oils and other secondary metabolites, including alkaloids, terpenoids, tannins and flavonoids [2,3]. The secondary metabolites of several plants have been found to be endowed with antimicrobial properties exerted through different mechanisms of action, such as the ability to form complexes with extracellular and soluble proteins, thus sequestering them, or with membrane proteins of targeted microbes, thus determining a perturbation of membrane organization. Some secondary metabolites have also been reported to cause membrane disruption and bacterial enzyme inhibition [4,5,6,7]. The abundance and bioactivities of these compounds may greatly vary depending on plant species and environmental conditions. Traditional herbal medicine is gaining great attention in developing countries due to the strong demand for natural compounds able to exert therapeutic effects in the absence of harmful side effects. However, it has to be highlighted that only a small percentage (1–10%) of available plant species is used by humans [8,9]. Furthermore, in many cases, the chemical composition of used plants has not been deeply characterized [3]. In recent years, the antimicrobial activity of several wild plants attracted the attention of scientists and researchers because of the fast spread of multidrug resistat (MDR) microorganisms, a worrying phenomenon requiring the urgent development of effective strategies alternative to conventional antibiotics [3]. 

Plants belonging to Ephedra genera falling in the Ephedraceae family represent a group of perennial gymnosperms distributed in arid and semiarid regions all over the world [10,11,12,13]. They have been largely employed in the practice of complementary and alternative medicine [14], even if it is not clear whether these practices are supported by the plants’ effective pharmacological properties or if they are merely based on folklore [15]. In this scenario, researchers focused their efforts to search for and to identify novel effective drugs starting from still-unexplored folk medicinal wild plants [16]. In the case of Ephedra genera, the presence of five species, such as *E. foeminea*, *E. alata*, *E. aphyla*, *E. ciliata* and *E. fragilis* [17], has been observed. Alanda (*Ephedra foeminea* or *Ephedra campylopoda*) is the Arabic name for a low stalky Eurasian shrub from the Ephedraceae family, omnipresent in northern Palestine and across the southeastern Mediterranean. In Oriental Arab medicine, it is used to treat agitation and skin rash. Moreover, the aerial parts of various *Ephedra* species were proven to contain active alkaloids, such as phenylpropylamino, alkaloids, ephedrine and pseudoephedrine [18]. The phytochemical analysis of the aqueous, methanolic and ethanolic extracts of *E. foeminea* revealed the presence of different phytoconstituents, with the methanolic extract containing (i) high levels of phenols, carbohydrates, sterols/steroids, flavones and lignin; (ii) moderate levels of tannins, quinones, amino acids, cardiac glycosides and phlobatannins; (iii) low levels of resins, terpenoids, flavonoids, coumarins, reducing sugars and anthocyanins; and (iv) the absence of alkaloids, saponins, anthraquinones and fixed oils and lipids [19]. It has been also reported in other *Ephedra* species a high content of the alkaloids ephedrine and pseudoephedrine, which are known to be endowed with several pharmaceutical properties, being able to increase heartrate and blood pressure, to promote bronchodilatation and to affect the central nervous system [20]. Because of this, plants from *Ephedra* genera have been widely used throughout the history of humankind, having been employed as an ointment to improve wound healing or to treat bronchial asthma, chills, colds, coughs, edema, fever, allergies, syphilis and gonorrhea [21]. *Ephedra* extracts have even been used as a food supplement to enhance performance and weight loss until 2004 when they were banned by the Food and Drug Administration (FDA) because of several death events due to negative effects on the cardiac and cardiovascular systems [22]. In more recent studies, the ethanolic extracts of *E. foeminea* were found to be endowed with anticancer and antimicrobial properties [23,24]. However, while the alkaloid content of *Ephedra* species has been widely described, only a few papers focus on non-alkaloid content. Based on this, it appears promising to investigate the biological activities of these still-unexplored secondary metabolites that might represent a source of novel compounds with interesting bioactivities.

In this study, we performed a bioguided purification of *E. foeminea* extracts to isolate bioactive compounds that were identified by nuclear magnetic resonance (NMR) spectroscopy and mass spectrometry (MS) analyses. Following this experimental strategy, in addition to the well-known monoterpenoid phenols carvacrol and thymol, four acylated kaempferol glycosides namely kaempferol-3-*O*-α-L-(2″,4″-di-*E*-*p*-coumaroyl)-rhamnopyranoside, kaempferol-3-*O*-α-L(2″-*E*-*p*-coumaroyl,4″-*Z-p*-coumaroyl)-rhamnopyranoside, kaempferol-3-*O*-α-L(2″-*Z*-*p*-coumaroyl,4″-*E*-*p*-coumaroyl)-rhamnopyranoside and kaempferol-3-*O*-α-L(2″,4″-di-*Z*-*p*-coumaroyl)-rhamnopyranoside were also identified for the first time in *E. foeminea*. Furthermore, they were found to be endowed with strong antimicrobial properties and promising antibiofilm activity. 

## 2. Results

### 2.1. Antimicrobial and Antibiofilm Activity of E. foeminea Extracts 

**Figure 1:**
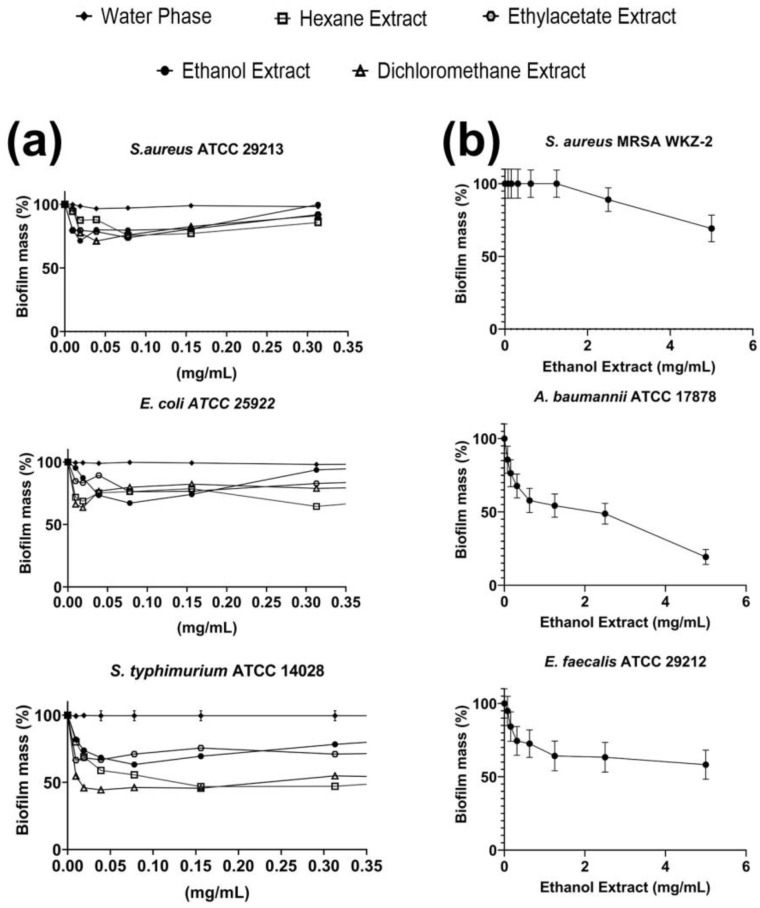
Antibiofilm activity of *E. foeminea* plant extracts on *S. aureus* ATCC 29213, *S. aureus* MRSA WKZ-2, *E. faecalis* ATCC 29212, *A. baumannii* ATCC 17878, *E. coli* ATCC 25922 and *S. typhimurium* ATCC 14028. (**a**) Extracts obtained in ethanol, hexane, dichloromethane, ethyl acetate or water phase were tested against *S. aureus* ATCC 29213, *E. coli* ATCC 25922 and *S. typhimurium* ATCC 14028. (**b**) Ethanolic extract was tested against *A. baumannii* ATCC 17878, *E. faecalis* ATCC 29212 and *S. aureus* MRSA WKZ-2. The effects of increasing concentrations of each extract were evaluated on biofilm formation. At the end of the incubations, biofilm samples were stained with crystal violet and measured at 630 nm. Data represent the mean (±standard deviation, SD) of at least three independent experiments, each one carried out with triplicate determinations.

*E. foeminea* ethanolic extract was firstly investigated for its antibacterial activity towards three gram-positive (*S. aureus* ATCC 29213, methicillin-resistant *S. aureus* MRSA WKZ-2, *E. faecalis* ATCC 29212) and three gram-negative (*A. baumannii* ATCC 17878, *E. coli* ATCC 25922 and *S. typhimurium* ATCC 14028) bacterial strains. The ethanolic extract was found to exert strong antimicrobial properties towards three out of six bacterial strains tested, with MIC_100_ values ranging from 1.25 to 2.5 mg/mL (Table 1). Being effective on both gram-negative and gram-positive bacterial strains, the ethanolic extract was found to be endowed with a broad-spectrum antimicrobial activity (Table 1). Interestingly, significant antimicrobial effects were also detected on *S. aureus* MRSA WKZ-2, *E. faecalis* ATCC 29212 and *A. baumannii* ATCC 17878 bacterial strains, even if higher doses of the extract (MIC_100_ values comprised between 5 and 10 mg/mL) were required to obtain the same effects recorded on the other tested strains (Table 1). To identify compounds responsible for the detected effects, further extraction procedures were sequentially performed as detailed in the Materials and Methods section. In particular, three solvents characterized by a different polarity, namely (i) hexane, (ii) dichloromethane and (iii) ethyl acetate were used. The antibacterial activity of each obtained fraction was investigated on the same bacterial strains described above and it was found that hexane, dichloromethane and ethyl acetate extracts were able to exert antimicrobial activity on three out of six bacterial strains tested with MIC_100_ values comprised between 0.625 and 2.5 mg/mL (Table 1). This suggests that the extracts retain a significant broad-spectrum antimicrobial activity being effective on both gram-negative and gram-positive bacterial strains (Table 1). In the case of the water phase, no significant antimicrobial activity was detected on any of the tested strains. Based on this, it can be hypothesized that most of the compounds responsible for the antibacterial activity were extracted using organic solvents. The antibiofilm properties of the extracts were also evaluated by performing crystal violet assays. Interestingly, ethanolic, hexane, dichloromethane and ethyl acetate extracts were found to be endowed with antibiofilm properties, being able to affect the biofilm formation in the case of *S. typhimurium* ATCC 14028 (Figure 1a). No significant antibiofilm properties were, instead, detected for the water phase (Figure 1a). Ethanolic extract was also tested on *S. aureus* MRSA WKZ-2, *E. faecalis* ATCC 29212 and *A. baumannii* ATCC 17878 and found to be effective on these strains with significant inhibition of biofilm formation (about 50–70% inhibition) in the case of *A. baumannii* ATCC 17878 and *S. aureus* MRSA WKZ-2 (Figure 1b).

### 2.2. Bioguided Fractionation of Plant Extracts Followed by Isolation and Identification of Active Compounds

**Figure 2:**
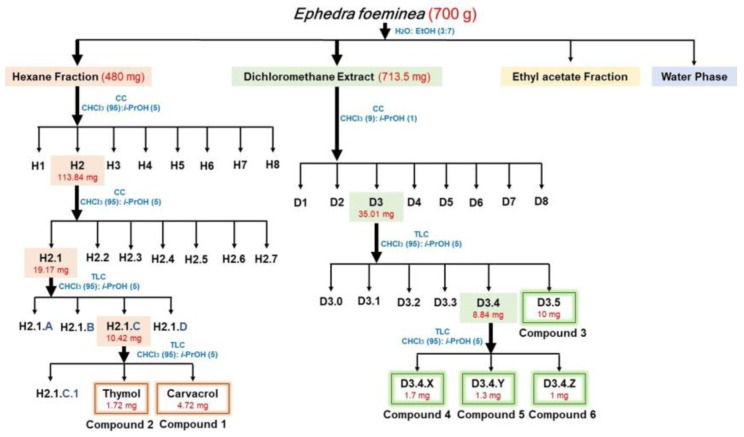
Schematic representation of the bioguided fractionation of extracts obtained from *E. foeminea* aerial parts.

To isolate the compounds endowed with antibacterial and/or antibiofilm activities, the most promising extracts (hexane and dichloromethane extracts) obtained from *E. foeminea* were subjected to sequential purification steps by thin-layer chromatography (TLC) or column chromatography (CC) as reported in the Materials and Methods section and in Figure 2. 

**Figure 3:**
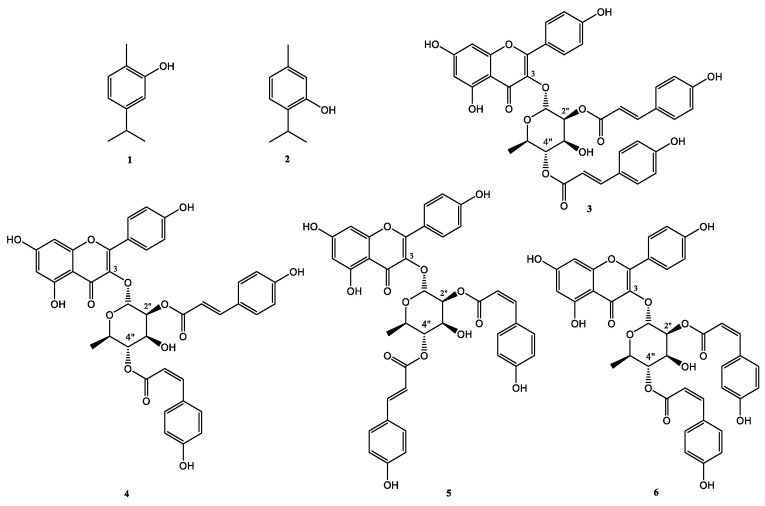
Chemical structures of isolated compounds **1**–**6**.

Fractions obtained upon extraction in hexane and two sequential steps of CC were found to be endowed with antimicrobial activity (Supplementary Table S1). Two main compounds were isolated from the hexane extract and identified as carvacrol (compound **1**, 4.72 mg) and as thymol (compound **2**, 1.72 mg) (Figure 3), comparing their spectroscopic data (^1^H NMR and ESI MS) with those reported in the literature (Supplementary Figures S1 and S2). It is noteworthy that no data in the literature exist about the presence of these two compounds in *E. foeminea* [21]. 

The active compounds from the CH_2_Cl_2_ extract were fractionated by combined CC and TLC on the direct and reverse phase to obtain four pure metabolites, as reported in detail in the Materials and Methods section. The first investigation of their ^1^H NMR spectra showed the signals of flavonol glycosides. In particular, the ^1^H NMR spectrum of compound **3** was recorded in acetone-*d*_6_ [25] and in CD_3_OD [26] (Supplementary Figures S3–S13). In acetone-*d*_6_, two broad signals at δ 6.28 (H-6) and 6.48 (H-8) were detected and the two doublet A_2_X_2_ aromatic system protons at δ 7.13 (H-3′,5′) and 7.91 (H-2′,6′), *J* = 8.7 Hz, typical of a kaempferol residue. The presence of the anomeric proton at δ 5.83 (H-1″, brs), the methine protons at δ 5.62 (H-2, brs), 4.20 (H-3, dd, *J* = 9.7 and 3.0 Hz), 4.98 (H-4, t, *J* = 9.7 Hz), 3.39 (dd, *J* = 9.7 and 6.1 Hz) and the secondary methyl group at δ 0.86 (H-6, d, *J* = 6.1 Hz) suggested the presence of an α-rhamnopyranoside moiety substituted in position H-2″ and H-4″ by two *p*-coumaroyl residues, as observed from their typical signals. In particular, the signals of the double bonds H-2′′′ and H-3′′′ at δ 6.44 and 7.68 (*J* = 16.0 Hz) and H-2′′′′ and H-3′′′′at δ 6.31 and 7.56 (*J* = 16.1 Hz) revealed the presence of two *E*-configured double bounds. COSY spectrum (Supplementary Figures S7 and S8) confirmed the connections around the rhamnopyranoside and the positions of the *p*-coumaroyl residues at H-2″ and H-4″of the sugar residue for the downfield shifts showed by these protons. Through an extensive study of 2D ^13^C NMR spectra (HSQC and HMBC), the chemical shifts were assigned to all the carbons and protons (Supplementary Figures S10–S13). In this way, compound **3** was identified as kaempferol-3-*O*-α-L-(2″,4″-di-*E*-*p*-coumaroyl)-rhamnopyranoside as previously reported [25,26,27,28]. The identification was also supported by the data collected by the electrospray ionization (ESI) MS spectrum, which showed the protonated [M + H]^+^ ion at *m*/*z* 725. 

Compounds **4**–**6** showed similar 1D and 2D ^1^H and ^13^C NMR spectra indicating that they differ from compound **3** for the configuration of the double bonds on the two *p*-coumaroyl residues (Supplementary Figures S14–S24). In particular, the ^1^H NMR spectrum of compound **4** differed from that of compound **3** for the signals of the double bonds H-2′′′ and H-3′′′ at δ 6.45 and 7.70 (*J* = 15.9 Hz) and H-2′′′′and H-3′′′′at δ 5.78 and 6.92 (*J* = 10.0 Hz), thus suggesting the presence of *trans*- and *cis*-*p*-coumaroyl moieties. Compound **4** was identified kaempferol-3-*O*-α-L-(2″-*E*-*p*-coumaroyl,4″-*Z*-*p*-coumaroyl)-rhamnopyranoside, as previously reported [28]. The ^1^H NMR spectrum of compound **5** differed from that of compound **3** for the signals of the double bonds H-2′′′ and H-3′′′ at δ 5.88 and 6.93 (*J* = 11.8 Hz) and H-2′′′′ and H-3′′′′ at δ 6.33 and 7.59 (*J* = 16.0 Hz), thus suggesting the presence of *trans*- and *cis*-*p*-coumaroyl moieties. Thus, compound **5** was identified as kaempferol-3-*O*-α-L-(2″-*Z*-*p*-coumaroyl,4″-*E*-*p*-coumaroyl)-rhamnopyranoside, as previously reported [29]. Compound **6** was, instead, identified as kaempferol-3-*O*-α-L-(2″,4″-di-*Z*-*p*-coumaroyl)-rhamnoside. In fact, its ^1^H NMR spectrum differed from that of compound **3** for the signals of the double bonds H-2′′′ and H-3′′′ at δ 5.77 and 6.87 (*J* = 12.0 Hz) and H-2′′′′ and H-3′′′′ at δ 5.89 and 6.92 (*J* = 12.0 Hz) [26]. Through extensive use of 1D and 2D ^1^H and ^13^C NMR spectra, the chemical shifts were assigned to all the carbons and protons of compounds **4**–**6** (Supplementary Figures S14–S24).

### 2.3. Evaluation of the Antimicrobial Activity of Isolated Compounds ***1**–**6***

**Figure 4:**
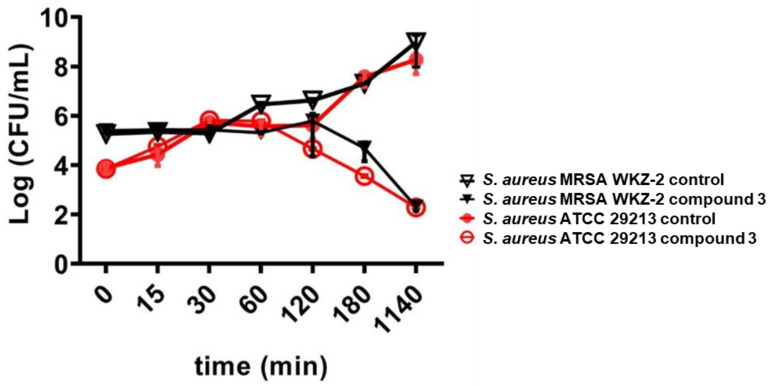
Time-killing curves obtained by incubating *S. aureus* ATCC 29213 (red lines) and *S. aureus* MRSA WKZ-2 (black lines) with kaempferol-3-*O*-α-L-(2″-*E*-*p*-coumaroyl,4″-*E*-*p*-coumaroyl)-rhamnoside (compound **3**) for different time intervals. Data represent the mean (±standard deviation, SD) of at least three independent experiments, each one carried out with triplicate determinations.

To verify whether the isolated compounds might account for the antimicrobial activity of the whole extract, antimicrobial activity assays were performed by testing increasing concentrations of each isolated compound on *S. aureus* ATCC 29213, *S. aureus* MRSA WKZ-2, *E. faecalis* ATCC 29212, *A. baumannii* ATCC 17878, *E. coli* ATCC 25922 and *S. typhimurium* ATCC 14028. As shown in Supplementary Table S2, carvacrol was found to be active on all the strains tested, with the strongest effects observed on *S. aureus* ATCC 29213 and *S. aureus* MRSA WKZ-2, with MIC_100_ values of 100 and 50 µg/mL, respectively (Supplementary Table S2). The *E. coli* bacterial strain was found to be the least sensitive to carvacrol antimicrobial activity, with an MIC_100_ value of 200 µg/mL (Supplementary Table S2). In the case of thymol, higher MIC_100_ values were detected with respect to carvacrol on the same bacterial strains, with *S. typhimurium* found to be the most susceptible bacterial strain (MIC_100_ = 300 µg/mL) and *S. aureus* MRSA WKZ-2 found to be the least sensitive to thymol antimicrobial effects (MIC_100_ = 2400 µg/mL) (Supplementary Table S2). The antimicrobial activity was also tested for the isolated isomer kaempferol-3-*O*-α-L-(2″,4″-di-*E*-*p*-coumaroyl)-rhamnopyranoside compound **3** on *S. aureus* ATCC 29213, *S. aureus* MRSA WKZ-2, *E. coli* ATCC 25922, *E. faecalis* ATCC 29212, *E. coli* ATCC 25922, *S. typhimurium* ATCC 14028 and *A. baumannii* ATCC 17878. A very strong antimicrobial activity towards gram-positive *S. aureus* ATCC 20213 and *S. aureus* MRSA WKZ-2 was found, with MIC_100_ values as low as 0.49 µg/mL in the case of both bacterial strains (Table 2). Significantly higher MIC_100_ values were detected in the case of the other bacterial strains tested, with values comprised between 250 and 1000 µg/mL (Table 2 and Supplementary Table S3). Kinetic killing curves were also obtained by treating *S. aureus* ATCC 29213 and *S. aureus* MRSA WKZ-2 with 0.49 µg/mL compound **3** for different times (0–19 h). We observed that, in the case of both bacterial strains, about 90% cell death was observed upon 19 h incubation (Figure 4), thus indicating the ability of kaempferol-3-*O*-α-L-(2″,4″-di-*E*-*p*-coumaroyl)-rhamnopyranoside compound **3** to exert bactericidal effects. Very interesting is also the evidence that the analyzed compound **3** was the only one to be endowed with this strong antimicrobial activity on *S. aureus* strains. Indeed, the other tested isomers (compound **4**–**6**) were found to be endowed with MIC_100_ values higher than 1000 µg/mL when tested on *S. aureus* MRSA WKZ-2 (Supplementary Table S3). This might open interesting perspectives to the applicability of the isolated compound **3** in the biomedical field.

### 2.4. Evaluation of the Antibiofilm Activity of Kaempferol-3-O-α-L-(2″,4″-di-E-p-coumaroyl)-rhamnopyranoside (Compound **3**)

**Figure 5:**
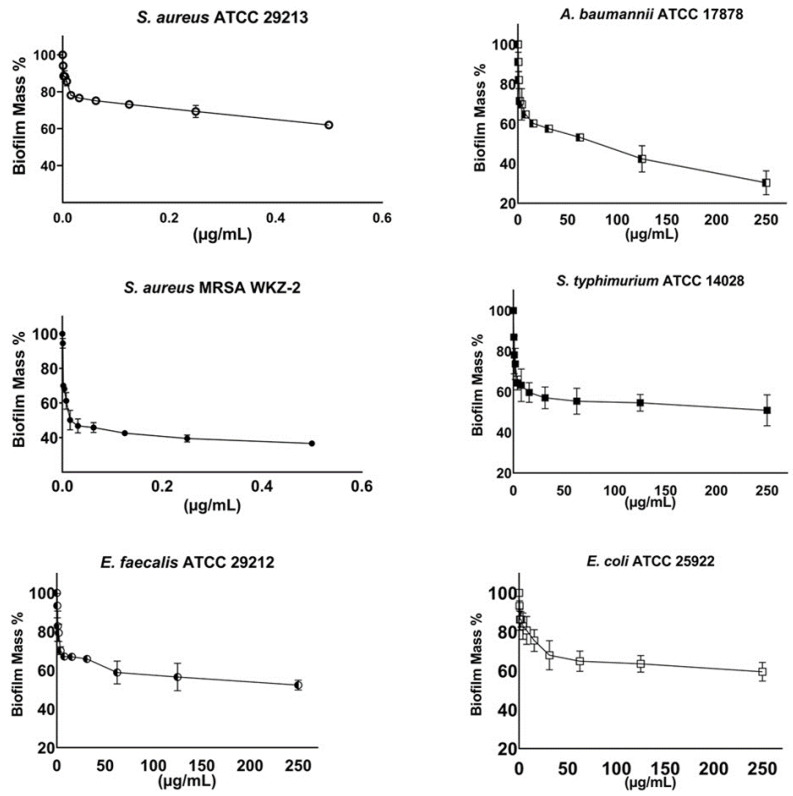
Antibiofilm activity of kaempferol-3-*O*-α-L-(2″,4″-di-*E-p*-coumaroyl)-rhamnoside (compound **3**) evaluated by crystal violet assays on *S. aureus* ATCC 29213, *S. aureus* MRSA WKZ-2, *E. faecalis* ATCC 29212, *A. baumannii* ATCC 17878, *S. typhimurium* ATCC 14028 and *E. coli* ATCC 25922. The effects of increasing concentrations of the compound were evaluated on biofilm formation. Biofilm was stained with crystal violet and samples were analyzed by using a plate reader to measure absorbance at 630 nm. Data represent the mean (±standard deviation, SD) of at least three independent experiments, each one carried out with triplicate determinations.

**Figure 6:**
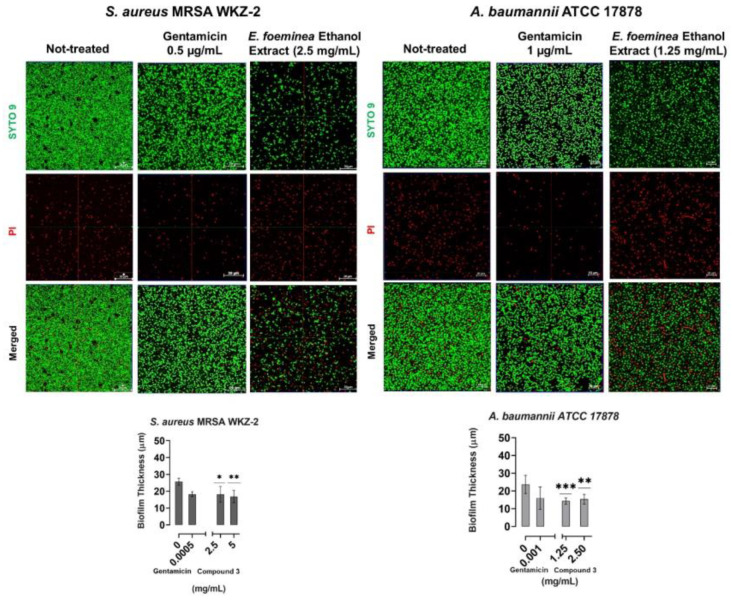
Antibiofilm activity of *E. foeminea* ethanolic extract. Effects of *E. foeminea* ethanolic extract were evaluated on biofilm formation in the case of *S. aureus* MRSA WKZ-2 and *A. baumannii* ATCC 17878 by CLSM. Gentamicin antibiotic was tested as a positive control. Not-treated sample contains the same amount of solvent present in the sample incubated with the highest concentration of ethanolic extract. Biofilm cells were stained with LIVE/DEAD BacLight bacterial viability kit (molecular probes, Eugene, OR) containing a 1:1 mol/mol ratio of SYTO-9 (green fluorescence, all cells) and propidium iodide (PI; red fluorescence, dead cells). The biofilm images were obtained by confocal z-stack using Zen Lite 2.3 software. All the images were taken under identical conditions. Significant differences were indicated as (* *p* < 0.05), (** *p* < 0.001) or (*** *p* < 0.0001) for treated versus control samples. Each experiment was carried out in triplicate.

**Figure 7:**
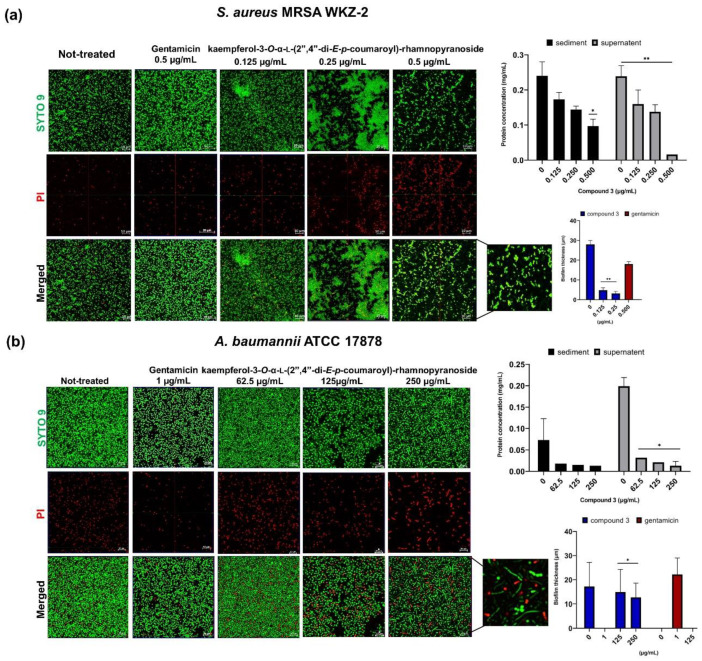
Antibiofilm activity of purified kaempferol-3-*O*-α-L-(2″,4″-di-*E-p*-coumaroyl)-rhamnopyranoside (compound **3**). Effects of compound **3** were evaluated on biofilm formation in the case of *S. aureus* MRSA WKZ-2 (**a**) and *A. baumannii* ATCC 17878 (**b**) by CLSM. Gentamicin antibiotic was tested as a positive control. Not-treated sample contains the same amount of solvent present in the sample incubated with the highest concentration of tested compound **3**. Biofilm cells were stained with LIVE/DEAD BacLight bacterial viability kit (molecular probes, Eugene, OR) containing a 1:1 mol/mol ratio of SYTO-9 (green fluorescence, all cells) and propidium iodide (PI; red fluorescence, dead cells). Biofilm images were obtained by confocal z-stack using Zen Lite 2.3 software. All the images were taken under identical conditions. Significant differences were indicated as (* *p* < 0.05) or (** *p* < 0.001) for treated versus control samples. Each experiment was carried out in triplicate.

The antibiofilm properties of the purified compound **3** were also evaluated by performing crystal violet assays on the *S. aureus* ATCC 29213, *S. aureus* MRSA WKZ-2, *E. faecalis* ATCC 29212, *A. baumannii* ATCC 17878, *E. coli* ATCC 25922 and *S. typhimurium* ATCC 14028 bacterial strains incubated with increasing concentrations of the isolated compound. In all the cases, a significant inhibition (about 30–80%) of biofilm formation was observed, with the strongest effects obtained in the case of the *A. baumannii* ATCC 17878 and *S. aureus* MRSA WKZ-2 bacterial strains (Figure 5). It is noteworthy that significant effects on biofilm formation were obtained at concentrations significantly lower than the MIC_100_ values detected on the same bacterial strains. To deepen on compound **3** antibiofilm activity, confocal laser scanning microscopy (CLSM) analyses were also performed. As first, the *E. foeminea* crude ethanolic extract was analyzed for its effects on the formation of *S. aureus* MRSA WKZ-2 and *A. baumannii* ATCC 17878 biofilm. As observed in Figure 6, significant effects on biofilm architecture and thickness were evaluated upon treatment of both bacterial strains with the extract for 24 h. When the pure compound **3** was analyzed on the same bacterial strains, significant effects on biofilm architecture and thickness were also evaluated (Figure 7). Furthermore, when the pure compound **3** was tested on *A. baumannii* ATCC 17878 biofilm, changes in the biofilm morphology were found to be associated with a strong aggregation of bacterial cells that appear to form groove-like structures (Figure 7a) and filamentous structures (Figure 7b), which is indicative of an interference with cell division, probably due to a septation block, as previously reported for different antibiofilm compounds [30]. A significant decrease in cell density also appears clear upon treatment with the pure compound (Figure 7), indicative of a high percentage of cell death. When the total protein content was analyzed, a significant effect of compound **3** was detected only in the case of the *S. aureus* MRSA WKZ-2 bacterial strain (Figure 7a). Indeed, in this case, the total protein content was found to be lower in the presence of compound **3** with respect to control untreated cells, both in the case of attached biofilm (sediment) and in the case of floating cells (supernatant). A different result was, instead, obtained in the case of the *A. baumannii* ATCC 17878 bacterial strain. In this case, a slight effect on the total protein content was detected only in the case of the supernatant (Figure 7b), probably because compound **3** exerts antibiofilm properties with different mechanisms depending on the specific features of the target bacterial cells. It is noteworthy that, when the antibiofilm activity of the isomers, i.e., compounds **4**–**6**, has been analyzed, different results were obtained. Also in this case, CLSM analyses were performed to test effects of isomers on *S. aureus* MRSA WKZ-2 and *A. baumannii* ATCC 17878 biofilm formation. In the case of compound **5**, a small effect on biofilm thickness was evaluated on *A. baumannii* ATCC 17878 upon 24 h incubation (Supplementary Figure S25), whereas no significant effects were detected on *S. aureus* MRSA WKZ-2 (Supplementary Figure S26). A similar result was obtained also in the case of the isomer compound **4** (Supplementary Figure S25), whereas no significant effects on either bacterial strain were detected in the case of compound **6** (Supplementary Figure S27).

### 2.5. Prediction of the Target of Kaempferol-3-O-α-L-(2″, 4″-di-E-p-coumaroyl)-rhamnopyranoside Antimicrobial and Antibiofilm Activity by Molecular Docking

**Figure 8:**
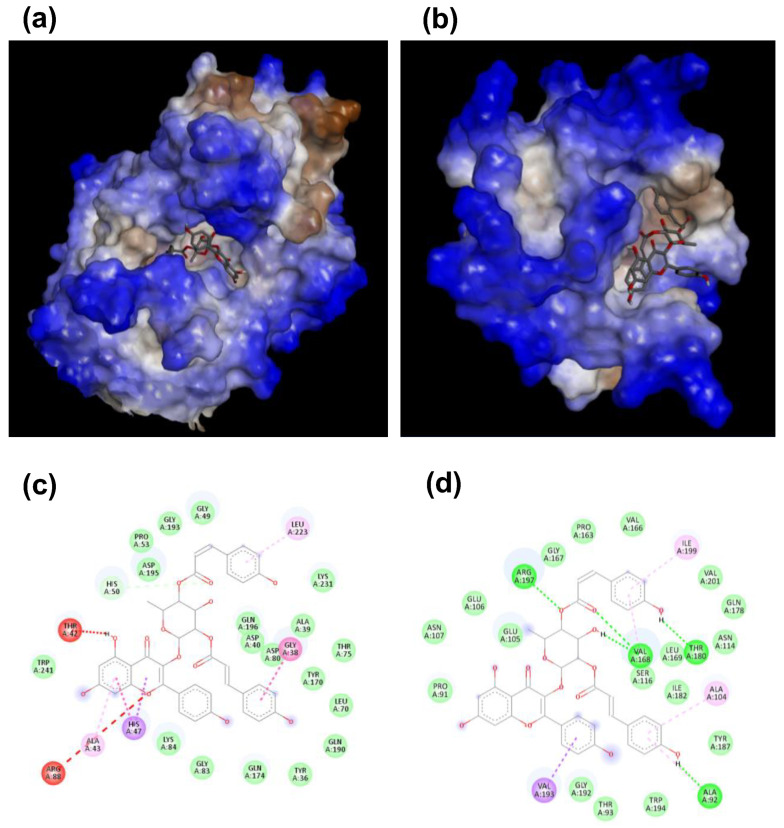
3D orientation of kaempferol-3-*O*-α-L-(2″,4″-di-*E*-*p*-coumaroyl)-rhamnopyranoside (compound **3**) (shown in stick) at the active pocket of tyrosyl tRNA synthetase (**a**) and Sortase A (**b**); 2D representation of intermolecular interactions between the antimicrobial compound and enzyme targets tyrosyl tRNA synthetase (**c**) and Sortase A (**d**).

Among the well-known proteins responsible for bacterial biofilm adhesion, formation and aggregation, Sortase A (Srt A) [31] attracted our attention. This enzyme catalyzes the covalent linkage of the cell wall proteins (CWA) to the peptidoglycan [31]. Indeed, the inactivation of their enzymatic activity has been reported to interfere with the ability of bacterial cells to cause infections because of the failure of fibronectin protein attachment to the cell wall [32]. For this reason, the Srt A protein attracted the attention of the researchers as an interesting target for the development of antimicrobial and antibiofilm drugs [33]. Another interesting target is represented by aminoacyl–tRNA synthases (aaRSs) [34] playing a key role in the translation of nucleic acid sequences into a polypeptide sequence [35]. Indeed, their inhibition is associated with a blockage of bacterial cell growth due to the interference with protein synthesis [34]. Furthermore, it has to be highlighted that significant differences in the topology of ATP binding domain have been reported between human and bacterial aaRS, probably due to the different functions that these enzymes play in human cells [36], thus opening the way to the possibility of selectively targeting bacterial aaRSs enzymes [34]. Based on this, a preliminary evaluation of the putative molecular bases of compound **3** antimicrobial and antibiofilm properties was performed by selecting Sortase A (Srt A) protein and aminoacyl–tRNA synthases (aaRSs) as targets in molecular docking analyses. The tested compound **3** demonstrated substantial binding to both selected targets. The binding free energy, in the case of the complex with tyrosyl tRNA synthetase, was computed to be higher (−9.5 kcal/mol) than that determined in the case of Sortase A (−8.3 kcal/mol). In the case of all the analyzed complexes, the binding of the ligand to the active site of the proteins was found to be mediated by representative hydrogen bonding, hydrophobic and various van der Waal’s force interactions (Supplementary Table S4, Figure 8a–d). Furthermore, in the case of the complex with tyrosyl tRNA synthetase, the binding was found to be supported by a carbon-hydrogen bond and several hydrophobic interactions. Indeed, the tested ligand was found to be bonded to several amino acid residues, such as Gly 38, Ala 43, His 47, His 50 and Leu 223 (Supplementary Table S4). The maximum number of interactions was observed in the case of His 47 residue. It is noteworthy that the residues His 47 and His 50 were reported as suitable targets of specific inhibitors of the enzyme tyrosyl tRNA synthetase [35]. In the case of the complex with Sortase A, various non-covalent forces were described including conventional hydrogen bonding, carbon–hydrogen bonding and hydrophobic interactions. Amino acids Ala 92, Ala 104, Val 168, Thr 180, Val 193, Arg 197 and Ile 199 were predicted to mediate the interaction between the test ligand and the Sortase A enzyme (Supplementary Table S4). It is noteworthy that residue Arg 197 represents a suitable target f an inactivating drug, since it has been reported to play a crucial role in the active site of the Sortase A enzyme [33]. It has to be highlighted that non-covalent forces generally mediate the biological activity of effective drugs and are of great importance in the design and development of novel drugs. Hydrogen bonds generally deliver structural strength and stability to protein–ligand complexes, whereas hydrophobic forces play a crucial role in amplifying the ligand’s binding affinity toward the surface of the target in a physiological environment [37,38]. Van der Waal’s interactions, being weak electrostatic forces, play an important role in stabilizing the three-dimensional structure of protein–ligand complexes [39]. Based on our findings, it can be hypothesized that the strong antibacterial activity of kaempferol-3-*O*-α-L-(2″,4″-di-*E*-*p*-coumaroyl)-rhamnoside against *S. aureus* bacterial cells might be mediated by its binding to these two selected enzymes (tyrosyl tRNA synthetase and Sortase-A) and to the consequent blockage of key residues present in the active site determining the inhibition of the enzymatic activity. 

### 2.6. Analysis of Kaempferol-3-O- α-L-(2″,4″-di-E-p-coumaroyl)-rhamnopyranoside Biocompatibility 

**Figure 9:**
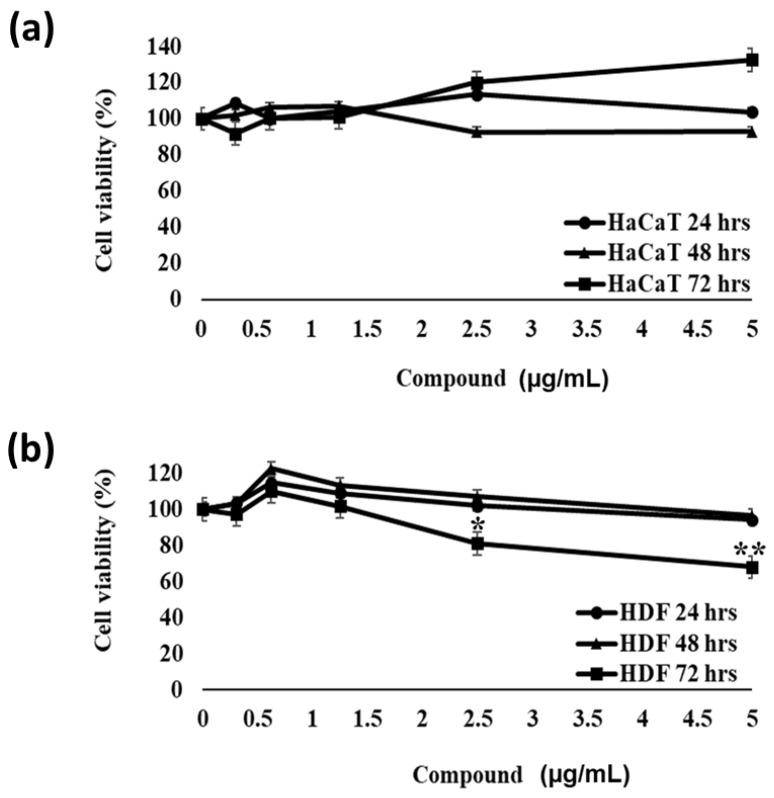
Effect of kaempferol-3-*O*-α-L-(2″,4″-di-*E-p*-coumaroyl)-rhamnopyranoside (compound **3**) on the viability of HaCat (**a**) and HDF cells (**b**). Cell viability was expressed as the percentage of MTT reduction with respect to control cells tested under the same conditions but in the absence of the compound under test. The experimental data represent the average of three independent experiments, each one carried out with triplicate determinations. Significant differences were indicated as (* *p* < 0.05) or (** *p* < 0.001) for treated versus control samples. Each experiment was carried out in triplicate.

To verify whether compound **3** satisfies the requirements to be employed in the future as a bioactive molecule in the biomedical field, biocompatibility analyses on eukaryotic cells were performed. For this purpose, immortalized human keratinocytes (HaCaT) and human dermal fibroblasts (HDF) were incubated with increasing concentrations (from 0.5 μg/mL to 5 μg/mL) of the compound under test for 72 h. As shown in Figure 9a,b, no significant toxic effects were detected under the experimental conditions tested, thus indicating that the identified compound is selectively toxic toward prokaryotic cells. Slight toxicity was detected only in the case of HDF cells at the highest concentrations tested (Figure 9b).

## 3. Discussion

In the present manuscript, a bioguided purification of *E. foeminea* extracts was carried out to isolate compounds with antimicrobial and antibiofilm properties. A total of six compounds were isolated and identified as carvacrol, thymol and four acylated kaempferol glycosides, i.e., kaempferol-3-*O*-α-L-(2″,4″-di-*E*-*p*-coumaroyl)-rhamnopyranoside, kaempferol-3-*O*-α-L-(2″-*E*-*p*-coumaroyl,4″-*Z-p*-coumaroyl)-rhamnopyranoside, kaempferol-3-*O*-α-L-(2″-*Z*-*p*-coumaroyl,4″-*E*-*p*-coumaroyl)-rhamnopyranoside and kaempferol-3-*O*-α-L-(2″,4″-di-*Z*-*p*-coumaroyl)-rhamnopyranoside (compounds **1**–**6** in Figure 3). These metabolites were identified for the first time in *E. foeminea* organic extracts and compounds **1**–**3** were found to be endowed with strong antimicrobial properties and promising antibiofilm activity.

Carvacrol and thymol have been largely investigated and are known for their medical properties. They have also been employed as food preservatives and food additives because of their well-known antimicrobial activity [40], probably due to their physicochemical properties, such as hydrophobicity, partition coefficient and ability to form H-bonds with interacting target molecules [41]. Carvacrol is an aromatic monoterpene generally present in aromatic plants such as thyme and oregano, and thymol is an isomer of carvacrol. Carvacrol has been reported to be endowed with significant antimicrobial properties when tested on a wide range of bacterial strains such as *E. coli*, *S. aureus*, *L. monocytogenes* and *S. typhimurium* [42,43,44,45]. Molecular mechanisms underlying carvacrol antimicrobial activity imply bacterial membrane disruption due to an alteration of its fluidity, integrity and functionality [40,41]. Carvacrol has been also reported to cause a depletion of intracellular ATP due to an alteration of intracellular pH value consequent to an interference with protons influx [40,41]. In the literature, further mechanisms have been also described to explain carvacrol effects on the viability of bacterial cells, such as the induction of reactive oxygen species (ROS) and the inhibition of efflux pumps [40,41]. Due to its hydrophobicity, carvacrol has also been reported to penetrate the hydrophobic environment of bacterial biofilm matrix, to interact with bacterial membranes and to interfere with their ability to form biofilms by altering the motility of bacterial cells and by reducing the cells’ ability to adhere to substrates [40,41]. Carvacrol antibiofilm activity has been also reported to be mediated by its ability to interfere with signaling pathways of quorum sensing (QS) by blocking the expression of specific involved genes [40,41]. Based on these observations, it is plausible that carvacrol might be, at least in part, responsible for the antimicrobial and antibiofilm activity observed by testing *E. foeminea* hexane extract (Table 1 and Figure 5).

We also identified for the first time in the *E. foeminea* dichloromethane extract four glycosilated isomers of kaempferol (compounds **3**–**6** in Figure 3). It has to be highlighted that compound **3** was isolated for the first time from *Pentachondra pumila* collected in New Zealand [25] and from unripe fruits of *Ocotea vellosiana* [46]. Subsequently, it was isolated from several sources, such as the leaves of *Laurus nobilis* [26], the buds of *Mammea longifolia* [27], the leaves of *Eriobotrya japonica* [47], the leaves of *Cinnamomun kotoense* [29], the aerial parts of *Epimedium sagittatum* and the leaves of *Machilus philippinens* [48]. Compound **4** was isolated for the first time from the aerial parts of *Epimedium sagittatum* together with compounds **3** and **5** and other related compounds [28]. Regarding compound **5**, it was isolated for the first time together with compound **3** from the leaves of *Cinnamomun kotoense*, and a mixture of both compounds was found to be able to suppress peripheral blood mononuclear cell (PBMC) production induced by phytohemagglutinin (PHA) [29]. Finally, compound **6** was isolated for the first time from several sources together with other isomers, such as from the leaves of *Laurus nobilis* [26] together with compound **3**, from the leaves of *Machilus philippinens* together with compound **4** [48] and from the leaves of *Eriobotrya japonica* together with compound **3** [47]. Very interestingly, here, we found that, despite other isomers, compound **3** is the only one to be endowed with a very strong antimicrobial activity towards *S. aureus* strains sensitive or resistant to conventional antibiotics (Table 2). In the past, strong antimicrobial properties have been reported for compounds **3** and **4** isolated from *Laurus nobilis* against methicillin-resistant *S. aureus* strains and against vancomycin-resistant Enterococci [49]. Considering the strong antibacterial activity of compound **3** (Table 2) with respect to that shown by its isomers (compounds **4–6** in Table S3), it is plausible that stereochemistry of the double bonds of the *p*-coumaroyl residues is a key structural feature associated to the antibiotic activity. In particular, the *E,E* stereochemistry of the double bonds of the *p*-coumaroyl residues seems to be fundamental (Table 2). 

As compound **3** was the most promising antimicrobial agent among the identified compounds, we also performed docking studies, with the main aim being to predict suitable molecular targets of compound **3** antibiofilm activity. Obtained results suggest that the antibiofilm activity of the test ligand against *S. aureus* strains might be correlated to the inhibition of Sortase A and/or tyrosyl tRNA synthetase. Sortase A (Srt A) is one of the well-known proteins responsible for biofilm adhesion, formation and aggregation [31]. This enzyme catalyzes the formation of covalent bonds between cell wall proteins (CWA) and peptidoglycan molecules of the bacterial cell wall [31]. It has been reported that, in the presence of inactivating mutations of Srt A, bacterial cells are no longer able to cause infections because of the failure of the attachment of fibronectin proteins to the cell wall [32]. This makes Srt A a suitable target for the development of novel and effective antibiofilm drugs [33]. Aminoacyl–tRNA synthases (aaRSs) also represent good candidates as targets of antimicrobial strategies [34]. Indeed, their inhibition is associated with a blockage of cell growth due to interference with protein synthesis processes [34]. Furthermore, differences in the topology of the ATP binding domain between human and bacterial enzymes are the basis of the possibility to selectively target bacterial enzymes [36]. Indeed, this is in line with the data obtained here by treating human eukaryotic cells with kaempferol-3-*O*-α-L-(2″,4″-di-*E*-*p*-coumaroyl)-rhamnopyranoside. Indeed, compound **3**, while exerting toxic effects on *S. aureus* bacterial cells, was found to be harmless towards immortalized human keratinocytes (HaCaT) and human dermal fibroblasts (HDF) at concentrations effective on bacterial cells under the experimental conditions tested (Figure 8).

Altogether, obtained data open interesting perspectives to the future applicability of the identified compound **3** in several fields to counteract bacterial infections. In the future, it the possibility of using this compound in combination with conventional antibiotics will be also explored, in order to develop effective combinatorial therapeutic approaches with the main aim of counteracting the worrying phenomenon of multidrug resistance. 

## 4. Materials and Methods

### 4.1. Materials

All the reagents were purchased from Sigma-Merck (Milan, Italy), unless specified otherwise. Optical rotations were measured on a Jasco P-1010 digital polarimeter (Tokyo, Japan); ^1^H and ^13^C nuclear magnetic resonance (NMR) spectra were recorded at 400 and 100 MHz, respectively, in CDCl_3_, acetone-*d*_6_ and CD_3_OD on a Bruker spectrometer (Billerica, MA, USA). The same solvents were used as internal standards. DEPT, COSY-45, HSQC, HMBC and NOESY experiments [50] were performed using Bruker microprograms. Electrospray ionization mass spectra (ESIMS) were performed using the LC/MS TOF system AGILENT 6230B (Agilent Technologies, Milan, Italy). Analytical and preparative thin layer chromatography (TLC) were performed on silica gel plates (Kieselgel 60, F_254_, 0.25 and 0.5 mm respectively) or on reverse phase (Kieselgel 60 RP-18 F_254_, 0.20 mm) plates and the compounds were visualized by exposure to UV light and/or iodine vapors and/or by spraying firstly with 10% H_2_SO_4_ in MeOH and then with 5% phosphomolybdic acid in EtOH, followed by heating at 110 °C for 10 min. Column chromatography (CC) was performed using silica gel (Merck, Kieselgel 60, 0.063–0.200 mm).

### 4.2. Ephedra Foeminea Plant Collection and Identification

The aerial parts of *E. foeminea* were collected from their natural habitat in West Bank-Palestine during September 2020. Identification of the plant was carried out at the Department of Biology and Biotechnology, An-Najah National University, Palestine. Representative plant specimens were collected, pressed until dried, then chemically poisoned to prevent bacterial and fungal infections and finally mounted on herbarium sheets and provided with a voucher number (ANUH1895). Subsequently, they were deposited at An-Najah National University herbarium. The aerial parts of *E. foeminea* collected were washed with water to remove soil and dust particles and then dried. Exposure to light was avoided to prevent possible loss of effective ingredients. The dried aerial parts were made into a fine powder using a blender in order to make them ready for subsequent extraction processes.

### 4.3. Plant Extract Preparation and Purification

Starting with the plants collected from their natural habitat in West Bank-Palestine during September 2020 (voucher number ANUH1895), plant material (700 g) was extracted (1 × 2000 mL) using ethanol/H_2_O (7/3, *v*/*v*) under stirring conditions at room temperature for 48 h. Afterward, the sample was centrifuged at 7000 rpm for 40 min. An amount of 10 mL of the supernatant was concentrated under reduced pressure in order to evaporate the ethanol and lyophilized to obtain 120 mg of ethanol extract. The rest of the supernatant was firstly extracted by hexane (3 × 800 mL), then with CH_2_Cl_2_ (3 × 800 mL) and, after removing the ethanol under reduced pressure, with EtOAc (3 × 700 mL). Each kind of extract and the residual water phase were then tested for antimicrobial properties on six bacterial strains. Since hexane organic extract (280 g) displayed interesting antibacterial activity, it was purified using column chromatography (CC) and eluted with CHCl_3_/i-PrOH (95/5, *v*/*v*), thus obtaining eight homogeneous fractions (H1–H8). Among them, fraction H2 was found to retain antibiotic activity and was further purified by using different steps of CC and TLC (Figure 2). Briefly, fraction H2 (113.8 mg) was purified by CC and eluted with ethyl acetate/hexane (40/60, *v*/*v*). The first obtained fraction (H2.1 in Figure 2) was further purified using two steps of TLC and eluted with CHCl_3_/i-PrOH (98/2, *v*/*v*), to obtain four homogeneous fractions. Among these, fraction H2.1.C (10.42 mg) was further purified to obtain a pure oil identified as carvacrol (compound **1**, 4.7 mg) and a pure amorphous solid identified as thymol (compound **2**, 1.7 mg).

The organic extract obtained in CH_2_Cl_2_ (713.5 mg) was found to retain interesting antimicrobial properties and was purified using CC and eluted with CH_2_Cl_2_/i-PrOH (9/1, *v*/*v*), thus obtaining eight homogeneous fractions (D1–D8) (Figure 2). Among them, fraction D3 (35.01 mg) was found to retain antibiotic activity and was further purified using two steps of TLC (Figure 2). Briefly, fraction D3 was purified using TLC and eluted in CHCl_3_/i-PrOH (9/1, *v*/*v*), thus obtaining six homogeneous fractions (Figure 2). Fraction D3.5 (10.06 mg) in Figure 2 was found to be a pure yellow powder identified as kaempferol-3-O-α-L-(2″,4″-di-*E*-*p*-coumaroyl)-rhamnopyranoside (compound **3**, 10.06 mg). Fraction D3.4 (8.84 mg) in Figure 2 was further purified using TLC to obtain three pure amorphous solids, fractions D3.4.X, D3.4.Y and D3.4.Z in Figure 2. The fractions were analyzed using NMR and mass spectrometry and identified as kaempferol-3-O-α-L-(2″-E-p-coumaroyl,4″-Z-p-coumaroyl)-rhamnopyranoside (compound **4**, 1.65 mg), kaempferol-3-O-α-L-(2”-*Z*-*p*-coumaroyl,4″-*E*-*p*-coumaroyl)-rhamnopyranoside (compound **5**, 1.56 mg) and kaempferol-3-O-α-L-(2″,4″-di-*Z*-*p*-coumaroyl)-rhamnopyranoside (compound **6**, 0.88 mg), respectively.

### 4.4. Bacterial Strains and Growth Conditions

A total of six bacterial strains were used in the present study, i.e., *S. aureus* ATCC 29213, methicillin-resistant *S. aureus* (MRSA WKZ-2), *E. faecalis* ATCC 29212, *A. baumannii* ATCC 17878, *E. coli* ATCC 25922 and *S. typhimurium* ATCC 14028. All the bacterial strains were grown in Muller Hinton Broth (MHB; Becton Dickinson Difco, Franklin Lakes, NJ, USA) and on Tryptic Soy Agar (TSA; Oxoid Ltd., Hampshire, UK). In all the experiments, the bacteria were inoculated and grown overnight in MHB at 37 °C. The next day, the bacteria were transferred to a fresh MHB tube and grown to the mid-logarithmic phase.

### 4.5. Antimicrobial Activity

The antimicrobial activity of *E. foeminea* extracts and its derived compounds were tested against six bacterial strains, i.e., *S. aureus* ATCC 29213, methicillin-resistant *S. aureus* (MRSA WKZ-2), *E. faecalis* ATCC 29212, *A. baumannii* ATCC 17878, *E. coli* ATCC 25922 and *S. typhimurium* ATCC 14028 by using the broth microdilution method [51,52]. All the bacterial strains were obtained from ATCC (American Type Culture Collection), with the only exception of *S. aureus* (MRSA WKZ-2), which was kindly provided by Dr. E.J.A. Veldhuizen from Utrecht University, Netherlands. The bacteria were grown to the mid-logarithmic phase in MHB at 37 °C and then diluted to 2 × 10^6^ CFU/mL in nutrient broth (NB, Difco, Becton Dickinson, Franklin 12 Lakes, NJ). To perform the assay, the bacterial samples were mixed 1:1 *v*/*v* with two-fold serial dilutions of the compound under test and incubated for 20 h at 37 °C. Following the incubation, each sample was diluted and plated on TSA in order to count the number of colonies. All the experiments were carried out in three independent replicates. The MIC_100_ values were determined as the lowest compound concentration responsible for no visible bacterial growth after overnight incubation. To kinetically analyze the antimicrobial activity of kaempferol-3-O-α-L-(2″,4″-di-E-p-coumaroyl)-rhamnoside (compound **3**), *S. aureus* ATCC 29213 and *S. aureus* MRSA WKZ-2, bacterial cells were grown overnight in the MHB medium at 37 °C. The bacteria were then diluted to 4 × 10^6^ CFU/mL in NB 0.5× and mixed with 0.5 μg/mL compound **3** (1:1 *v*/*v*) for defined time intervals. The samples (20 μL) were serially diluted (from 10- to 100,000-fold) and 100 μL of each dilution was plated on TSA. Following an incubation of 16 h at 37 °C, the bacterial colonies were counted [51].

### 4.6. Antibiofilm Activity Assays

To evaluate antibiofilm effects, bacteria inocula were grown overnight at 37 °C, then diluted to 1 × 10^8^ CFU/mL in 0.5× MHB containing increasing concentrations of the compound under test. The gentamicin antibiotic was tested as a positive control at a concentration of 0.5 or 1 µg/mL on the *S. aureus* (MRSA WKZ-2) and *A. baumannii* ATCC 17878 bacterial strains, respectively. Not-treated samples contained the same amount of solvent present in the sample incubated with the highest concentration of the tested compound. The samples were then incubated at 37 °C for 24 h in order to test the effects on biofilm formation. In the case of crystal violet assay, the bacterial biofilm was washed three times with phosphate buffer (PBS 1X) and then incubated with the dye (0.04%) for 20 min at room temperature. Following the incubation, the samples were washed with PBS and then the dye bound to cells was dissolved in 33% acetic acid. Spectrophotometric measurements were then carried out at a wavelength of 630 nm by using a microtiter plate reader (FLUOstar Omega, BMG LABTECH and Germany) [51,52,53]. Confocal laser scanning microscopy (CLSM) analyses in static conditions were carried out by using Thermo Scientific™ Nunc™ Lab-Tek™ Chambered Coverglass systems (Thermo Fisher Scientific, Waltham, MA, USA). The viability of the cells within the biofilm structure was evaluated using sample staining with a LIVE/DEAD^®^ Bacterial Viability kit (Molecular Probes Thermo Fisher Scientific, Waltham, MA, USA). The staining was performed according to the manufacturer’s instructions. Biofilm images were collected by using a confocal laser scanning microscope (Zeiss LSM 710, Zeiss, Germany) and a 63× objective oil-immersion system. The biofilm architecture was analyzed by using the Zen Lite 2.3 software package. Each experiment was performed in triplicate and all images were taken under identical conditions [54]. The total protein content was determined by Bradford assay. Briefly, bacterial cells were grown overnight in MHB at 37 °C. The bacteria were then diluted to 4 × 10^8^ CFU/mL in 100 μL of 0.5× MHB 0.5× along with increasing concentrations of kaempferol-3-O-α-L-(2″,4″-di-E-p-coumaroyl)-rhamnoside compound 3 (1:1 *v*/*v*). The samples were then incubated at 37 °C for 24 h. Afterward, the biofilm supernatant was separated from the pellet by centrifugation and the protein content of obtained samples was determined by Bradford assay [55].

### 4.7. Eukaryotic Cell Cultures and Biocompatibility Evaluation

The immortalized human keratinocytes (HaCaT) and human dermal fibroblasts (HDF) were from Thermo Fisher Scientific, Waltham, MA, USA. Both cell lines were cultured in high-glucose Dulbecco’s modified Eagle’s medium (DMEM) supplemented with 10% fetal bovine serum (FBS), 1% antibiotics (Pen/strep) and 1% L-glutamine and grown at 37 °C in a humidified atmosphere containing 5% CO_2_. To evaluate the biocompatibility of kaempferol-3-O-(2″,4″-di-E-p-coumaroyl)-α-L-rhamno-pyiranoside, the cells were seeded into 96-well plates at a density of 3 × 10^3^ cells/well in 100 µL of complete DMEM 24 h before the treatment. They were then incubated in the presence of increasing compound concentrations (0.5–5 µg mL^−1^) for 72 h. Following the treatment, the cell culture supernatants were replaced with 0.5 mg/mL MTT (3-(4,5-dimethylthiazol-2-yl)-2,5-diphenyltetrazolium bromide) reagent dissolved in a DMEM medium without red phenol (100 µL/well). After 4 h of incubation at 37 °C, the resulting insoluble formazan salts were solubilized in 0.01 N HCl in anhydrous isopropanol and quantified by measuring the absorbance at λ = 570 nm using an automatic plate reader spectrophotometer (Synergy™ H4 Hybrid Microplate Reader, BioTek Instruments, Inc., Winooski, VT, USA), as previously described. Cell survival was expressed as the mean of the percentage values compared to the control untreated cells.

### 4.8. Molecular Docking Analyses

The chemical structure of kaempferol-3-O-α-L-(2″,4″-di-*E*-*p*-coumaroyl)-rhamnopyranoside was prepared and used as a ligand in molecular docking analyses with the main aim to predict the ligand’s binding affinity and possible chemical interactions with two putative interactors present in *S. aureus* cells, i.e., Sortase A (PDB ID-1T2P) and tyrosyl tRNA synthetase (TyRS) (PDB ID- 1JIL). To do this, the crystallographic 3D structures of the target enzymes were retrieved from the protein data bank. The Autodock tool 1.5.6 was used to optimize the structures of the putative receptor and of the ligand. This was performed upon removal of the water molecules and heteroatoms and addition of polar hydrogens and Kollman charges. Molecular docking was performed by using the CB Dock web server, which represents an implementation of the popular docking program Autodock Vina [56]. The docked complexes were visualized to identify putative receptor– ligand interactions by using Discovery Studio (DS) Visualizer 2020 (Biovia, San Diego, USA). The binding affinity of the compound under test towards the targets was estimated on the basis of intermolecular interactions and the bonds’ lengths (Supplementary Table S4).

### 4.9. Statistical Analyses 

Statistical analyses were performed by using Student’s *t*-test. Significant differences were indicated as * (*p* < 0.05), ** (*p* < 0.01), *** (*p* < 0.001) or **** (*p* < 0.0001). Graphs were obtained by using GraphPad Prism 8 software.

## Figures and Tables

**Table 1:** MIC_100_ values (mg/mL) determined for *E. foeminea* ethanolic, hexane, dichloromethane and ethyl-acetate extracts tested on a panel of gram-positive and gram-negative bacterial strains. (-) indicates not tested samples. Reported data refer to three biological replicates.

MIC_100_ (mg/mL)				
Gram-Positive Strains	Ethanol	Hexane	Dichloromethane	Ethyl Acetate
*S. aureus* ATCC 29213	2.5	0.625	1.25	2.5
*S. aureus* MRSA WKZ-2	10	-	-	-
*E. faecalis* ATCC 29212	10	-	-	-
**Gram-Negative Strains**				
*E. coli* ATCC 25922	1.25	2.5	2.5	2.5
*S. typhimurium* ATCC 14028	2.5	1.25	2.5	2.5
*A. baumannii* ATCC 17878	5	-	-	-

**Table 2:** MIC_100_ values (µg/mL) determined for the compound kaempferol-3-*O*-α-L-(2″,4″-di-*E*-*p*-coumaroyl)-rhamnopyranoside (compound **3**) purified from *E. foeminea* dichloromethane extract.

MIC_100_ (µg/mL)		
Gram-Positive Strains	Compound 3	Gentamycin
*S. aureus* ATCC 29213	0.49	≤1
*S. aureus* MRSA WKZ-2	0.49	≤1
*E. faecalis* ATCC 29212	500	≤8
**Gram-Negative Strains**		
*E. coli* ATCC 25922	250	≤4
*S. typhimurium* ATCC 14028	1000	≤15
*A. baumannii* ATCC 17878	1000	≤2

## Data Availability

The data presented in this study are available within the article or supplementary material.

## Supplementary Materials

The following supporting information can be downloaded at: https://www.mdpi.com/article/10.3390/ijms24043284/s1.
