# Supplementary material for: Ephedra foeminea as a Novel Source of Antimicrobial and Anti-Biofilm Compounds to Fight Multidrug Resistance Phenotype
Source: Int J Mol Sci. 2023 Feb 7;24(4):3284. doi: 10.3390/ijms24043284 (PMC9965181; doi:10.3390/ijms24043284)
Supplement: Supplementary file 1 [file ijms-24-03284-s001.zip › ijms-2146957-supplementary.pdf]

# SUPPORTING INFORMATION

# *Ephedra foeminea* as a Novel Source of Antimicrobial and Anti-Biofilm Compounds to Fight Multidrug Resistance Phenotype

**Shurooq Ismail [1,2], Rosa Gaglione [1,3], Marco Masi [1], Srichandan Padhi [4], Amit K. Rai [4], Ghadeer Omar [2], Alessio Cimmino [1,*] and Angela Arciello [1,3,*]**

[1] Department of Chemical Sciences, University of Naples Federico II, Via Cintia 21, I-80126 Naples, Italy
[2] Department of Biology and Biotechnology, An-Najah National University, Nablus 97300, Palestine
[3] Istituto Nazionale di Biostrutture e Biosistemi (INBB), 00136 Rome, Italy
[4] Institute of Bioresources and Sustainable Development, Imphal, Manipur 795004, India
* Correspondence: alessio.cimmino@unina.it (A.C.); anarciel@unina.it (A.A.)

## Supporting information list

**Supplementary Table S1.** Minimal Inhibitory Concentration ($MIC_{100}$) values (mg/mL) determined for fractions obtained upon extraction in hexane and two sequential steps of column chromatography.

**Supplementary Figure S1**. $^1$H NMR spectrum of carvacrol, **compound 1** ($CDCl_3$, 400 MHz).

**Supplementary Figure S2**. $^1$H NMR spectrum of thymol, **compound 2** ($CDCl_3$, 400 MHz).

**Supplementary Figure S3**. $^1$H NMR spectrum of kaempferol-3-*O*-α-L-(2”,4”-di-*E*-*p*-coumaroyl)-rhamnoside, **compound 3** (MeOD, 400 MHz).

**Supplementary Figure S4**. $^1$H NMR spectrum of kaempferol-3-*O*-α-L-(2”,4”-di-*E*-*p*-coumaroyl)-rhamnoside, **compound 3** (MeOD, 400 MHz) in the range 8.1 to 5.4 ppm.

**Supplementary Figure S5**. $^1$H NMR spectrum of kaempferol-3-*O*-α-L-(2”,4”-di-*E*-*p*-coumaroyl)-rhamnoside, **compound 3** (acetone-$d_6$, 400 MHz).

**Supplementary Figure S6**. $^1$H NMR spectrum of kaempferol-3-*O*-α-L-(2”,4”-di-*E*-*p*-coumaroyl)-rhamnoside, **compound 3** (acetone-$d_6$, 400 MHz) in the range 8.0 to 5.5 ppm.

**Supplementary Figure S7**. COSY spectrum of kaempferol-3-*O*-α-L-(2”,4”-di-*E*-*p*-coumaroyl)-rhamnoside, **compound 3** (acetone-$d_6$, 400 MHz).

**Supplementary Figure S8**. COSY spectrum of kaempferol-3-*O*-α-L-(2”,4”-di-*E*-*p*-coumaroyl)-rhamnoside, **compound 3** (acetone-$d_6$, 400 MHz) in the range 8.0 to 5.6 ppm.

**Supplementary Figure S9**. NOESY spectrum of kaempferol-3-*O*-α-L-(2”,4”-di-*E*-*p*-coumaroyl)-rhamnoside, **compound 3** (acetone-$d_6$, 400 MHz).

**Supplementary Figure S10**. $^{13}$C NMR spectrum of kaempferol-3-*O*-α-L-(2”,4”-di-*E*-*p*-coumaroyl)-rhamnoside, **compound 3** (acetone-$d_6$, 400 MHz).

**Supplementary Figure S11**. HSQC spectrum of kaempferol-3-*O*-α-L-(2”,4”-di-*E*-*p*-coumaroyl)-rhamnoside, **compound 3** (acetone-$d_6$, 400 MHz).

**Supplementary Figure S12**. HMBC spectrum of kaempferol-3-*O*-α-L-(2”,4”-di-*E*-*p*-coumaroyl)-rhamnoside, **compound 3** (acetone-$d_6$, 400 MHz).

**Supplementary Figure S13**. ESI MS spectrum of kaempferol-3-*O*-α-L-(2”,4”-di-*E*-*p*-coumaroyl)-rhamnoside, **compound 3** recorded in positive modality.

**Supplementary Figure S14**. $^1$H NMR spectrum of kaempferol-3-*O*-α-L-(2”-*E*-*p*-coumaroyl,4”-*Z*-*p*-coumaroyl)-rhamnoside, **compound 4** (MeOD, 400 MHz).

**Supplementary Figure S15**. $^1$H NMR spectrum of kaempferol-3-*O*-α-L-(2”-*E*-*p*-coumaroyl,4”-*Z*-*p*-coumaroyl)-rhamnoside, **compound 4** (MeOD, 400 MHz) in the range 8.0 to 5.3 ppm.

**Supplementary Figure S16**. COSY spectrum of kaempferol-3-*O*-α-L-(2”-*E*-*p*-coumaroyl,4”-*Z*-*p*-coumaroyl)-rhamnoside, **compound 4** (acetone-$d_6$, 400 MHz).

**Supplementary Figure S17**. $^1$H NMR spectrum of kaempferol-3-*O*-α-L-(2”-*E*-*p*-coumaroyl,4”-*Z*-*p*-coumaroyl)-rhamnoside, **compound 4** (acetone-$d_6$, 400 MHz).

**Supplementary Figure S18**. $^1$H NMR spectrum of kaempferol-3-*O*-α-L-(2”-*E*-*p*-coumaroyl,4”-*Z*-*p*-coumaroyl)-rhamnoside, **compound 4** (acetone-$d_6$, 400 MHz) in the range 8.0 to 5.4 ppm.

**Supplementary Figure S19**. $^1$H NMR spectrum of kaempferol-3-*O*-α-L-(2”-*Z*-*p*-coumaroyl,4”-*E*-*p*-coumaroyl)-rhamnoside, **compound 5** (MeOD, 400 MHz).

**Supplementary Figure S20**. $^1$H NMR spectrum of kaempferol-3-*O*-α-L-(2”-*Z*-*p*-coumaroyl,4”-*E*-*p*-coumaroyl)-rhamnoside, **compound 5** (MeOD, 400 MHz) in the range 8.0 to 5.4 ppm.

**Supplementary Figure S21**. $^{1}H$ NMR spectrum of kaempferol-3-*O*-α-L-(2”,4”-di-*Z*-*p*-coumaroyl)-rhamnoside, **compound 6** (MeOD, 400 MHz).

**Supplementary Figure S22**. $^{1}H$ NMR spectrum of kaempferol-3-*O*-α-L-(2”,4”-di-*Z*-*p*-coumaroyl)-rhamnoside, **compound 6** (MeOD, 400 MHz) in the range 7.9 to 5.5 ppm.

**Supplementary Figure S23**. $^{1}H$ NMR spectrum of kaempferol-3-*O*-α-L-(2”,4”-di-*Z*-*p*-coumaroyl)-rhamnoside, **6** (acetone-$d_6$, 400 MHz).

**Supplementary Figure S24**. $^{1}H$ NMR spectrum of kaempferol-3-*O*-α-L-(2”,4”-di-*Z*-*p*-coumaroyl)-rhamnoside, **compound 6** (acetone-$d_6$, 400 MHz) in the range 8.0 to 5.4 ppm.

**Supplementary Table S2**. $MIC_{100}$ values (μg/mL) determined for the essential oils carvacrol and thymol purified from *E. foeminea* hexane extract.

**Supplementary Table S3.** $MIC_{100}$ values (μg/mL) determined for the compounds kaempferol-3-O-(2",4"-di-E-p-coumaryl)-α-L-rhamno-piranoside, kaempferol-3-O-(2"-Z-p-coumaryl,4"-di-E-p-coumaryl)-α-L-rhamno-piranoside, kaempferol-3-O-(2"-E- p-coumaryl,4"-di-Z-p-coumaryl)-α-L-rhamno-piranoside and kaempferol-3-O-(2",4"-di-Z-p-coumaryl)-α-L-rhamno-piranoside purified from *E. foeminea* dichloromethane extract.

**Supplementary Figure S25.** Anti-biofilm activity of purified kaempferol-3-O-(2”-Z-p-coumaroyl,4”-E-p-coumaroyl)-α-L-rhamno-piranoside.

**Supplementary Figure S26.** Anti-biofilm activity of purified kaempferol-3-O-(2”-E-p-coumaroyl,4”-Z-p-coumaroyl)-α-L-rhamno-piranoside.

**Supplementary Figure S27.** Anti-biofilm activity of purified kaempferol-3-O-(2”,4”-di-Z-p-coumaroyl)-α-L-rhamno-piranoside.

**Supplementary Table S4.** Details of the intermolecular interactions between the test ligand and *S. aureus* target enzymes tyrosyl tRNA synthetase and sortase A.

**Supplementary Table S1**. $MIC_{100}$ values (mg/mL) determined for fractions obtained upon extraction in hexane and two sequential steps of column chromatography.

| | $MIC_{100}$ (mg/mL) of fractions extracted in hexane | | | | | | |
|---|---|---|---|---|---|---|---|
| **Bacterial strains** | **1** | **2** | **3** | **4** | **5** | **6** | **7** |
| *S. aureus* ATCC 29213 | 2.5 | 2.5 | 2.5 | 0.313 | 0.313 | 2.5 | 1.25 |
| *E. coli* ATCC 25922 | 2.5 | 2.5 | 2.5 | 2.5 | 2.5 | 2.5 | 2.5 |
| *S. typhimurium* ATCC 14028 | 2.5 | 2.5 | 2.5 | 2.5 | 2.5 | 2.5 | 2.5 |

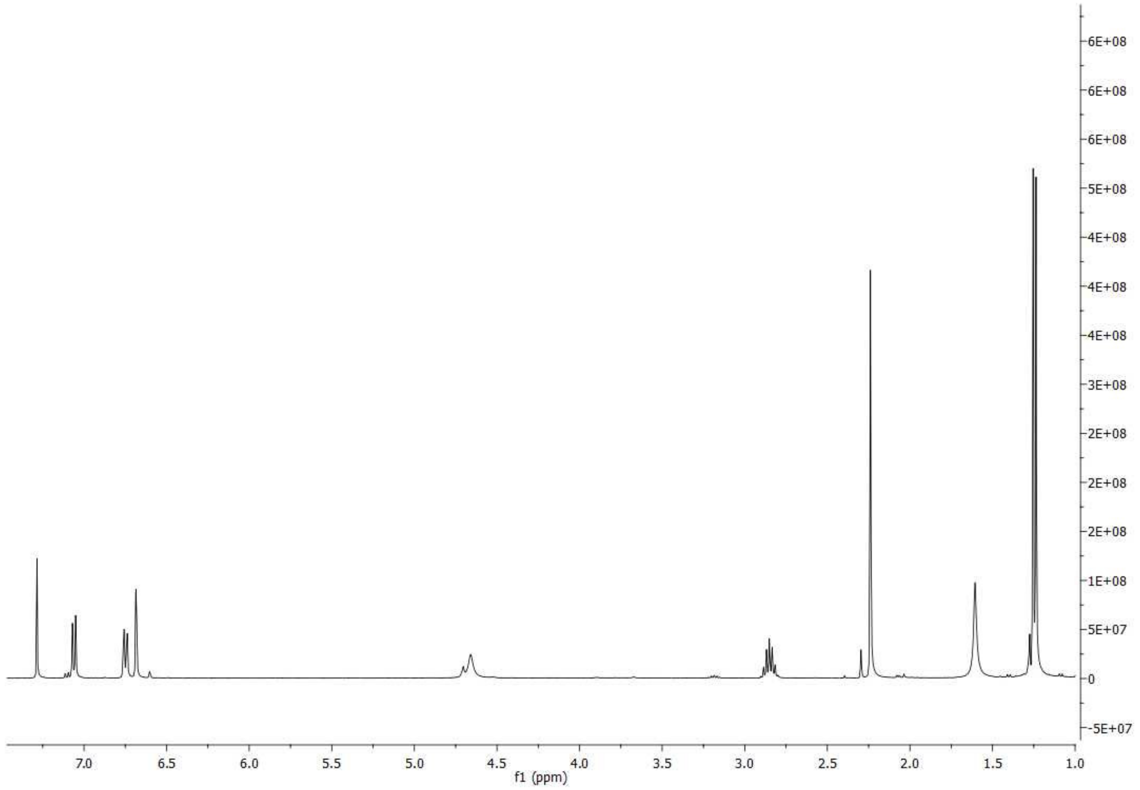


**Supplementary Figure S1**. $^{1}$H NMR spectrum of carvacrol, **compound 1** ($CDCl_3$, 400 MHz).

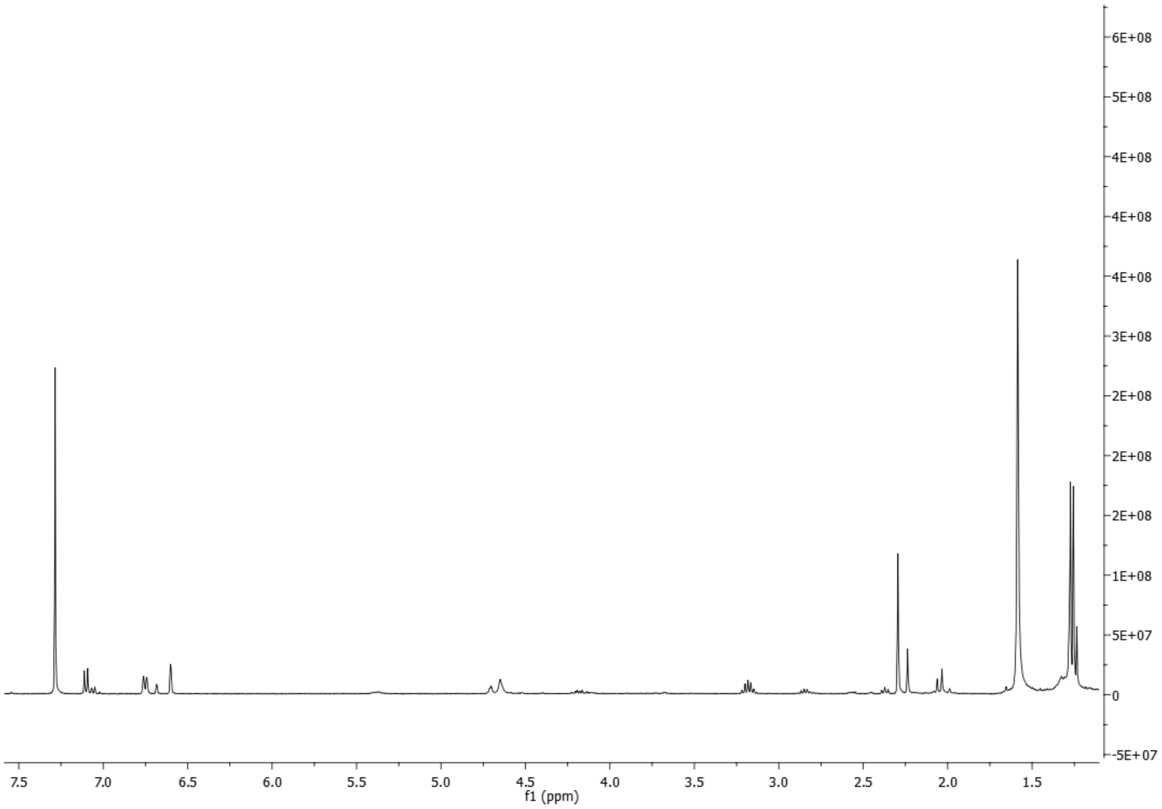


**Supplementary Figure S2**. $^{1}$H NMR spectrum of thymol, **compound 2** ($CDCl_3$, 400 MHz).

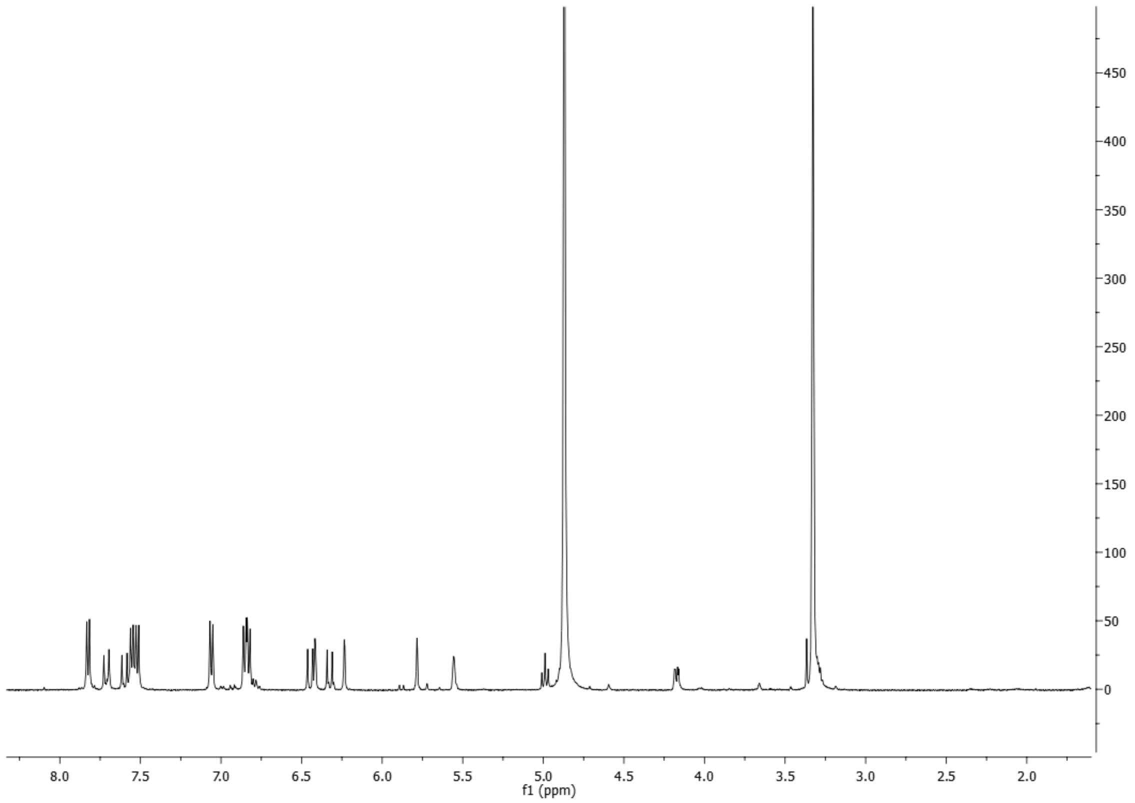


**Figure S3**. $^{1}H$ NMR spectrum of kaempferol-3-*O*-α-L-(2”,4”-di-*E*-*p*-coumaroyl)-rhamnoside, **compound 3** (MeOD, 400 MHz).

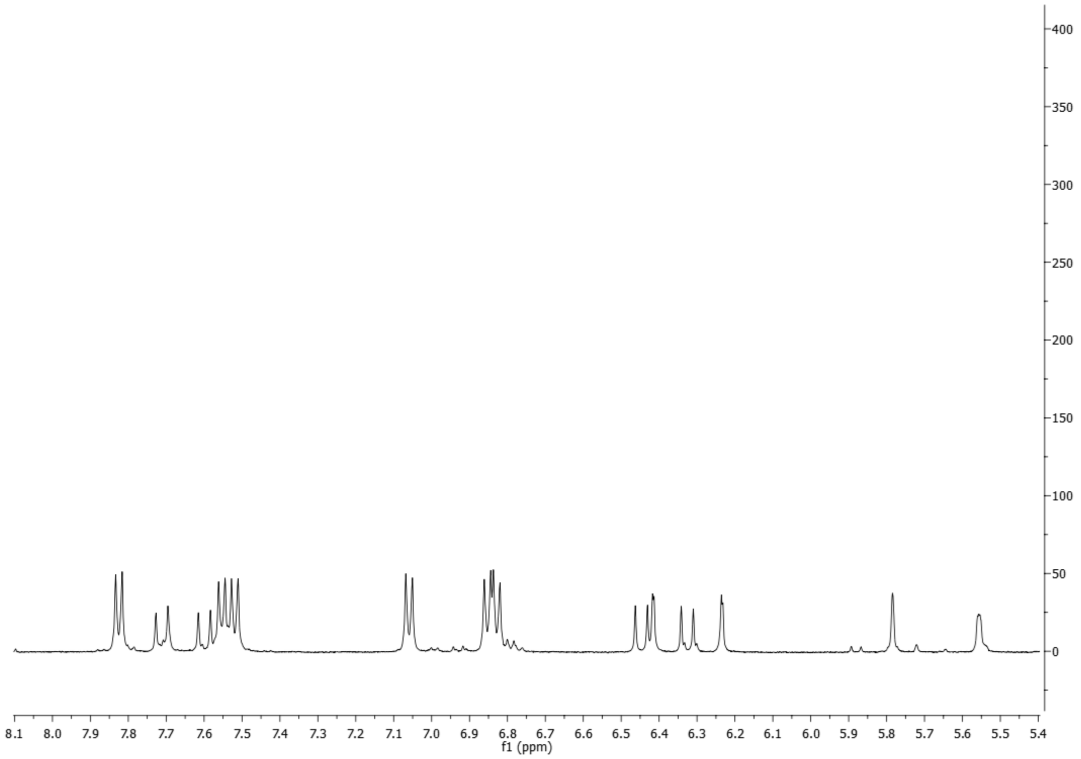


**Figure S4**. $^{1}H$ NMR spectrum of kaempferol-3-*O*-α-L-(2”,4”-di-*E*-*p*-coumaroyl)-rhamnoside, **compound 3** (MeOD, 400 MHz) in the range 8.1 to 5.4 ppm.

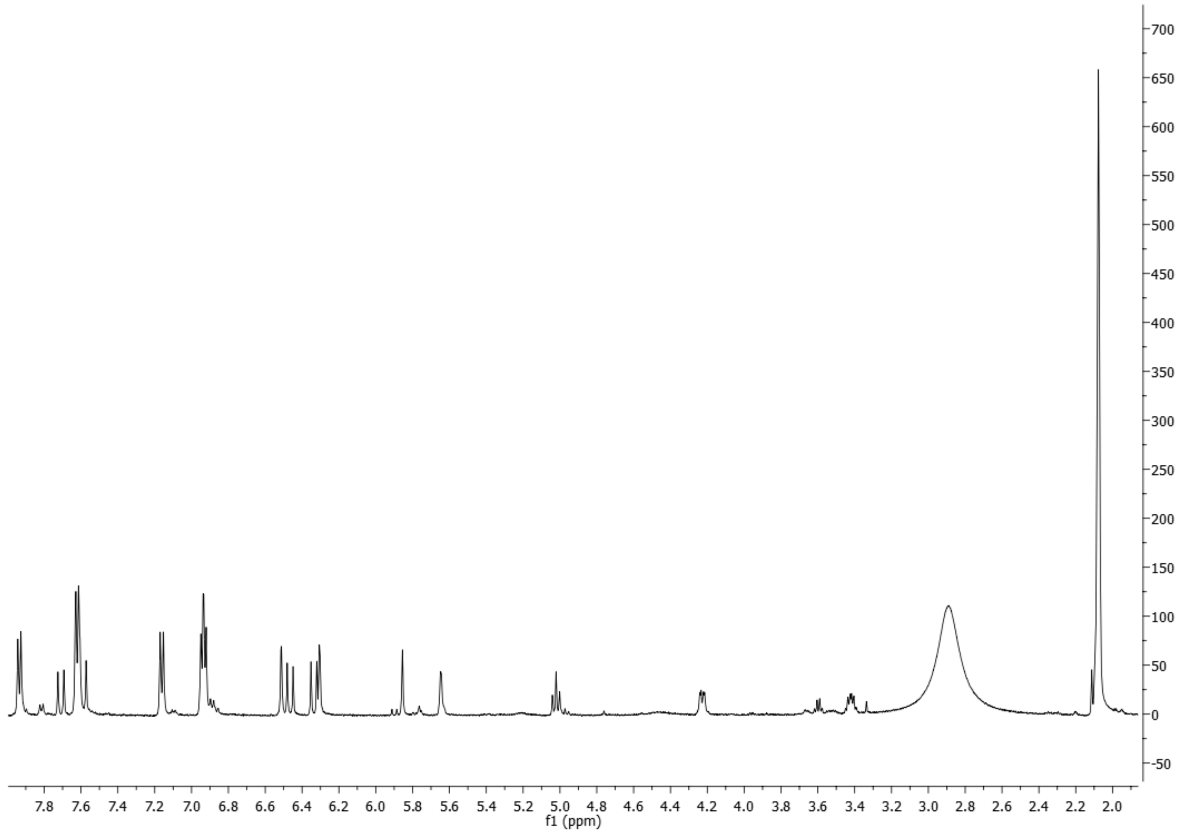


**Figure S5**. $^{1}$H NMR spectrum of kaempferol-3-*O*-α-L-(2”,4”-di-*E*-*p*-coumaroyl)-rhamnoside, **compound 3** (acetone-*d*6, 400 MHz).

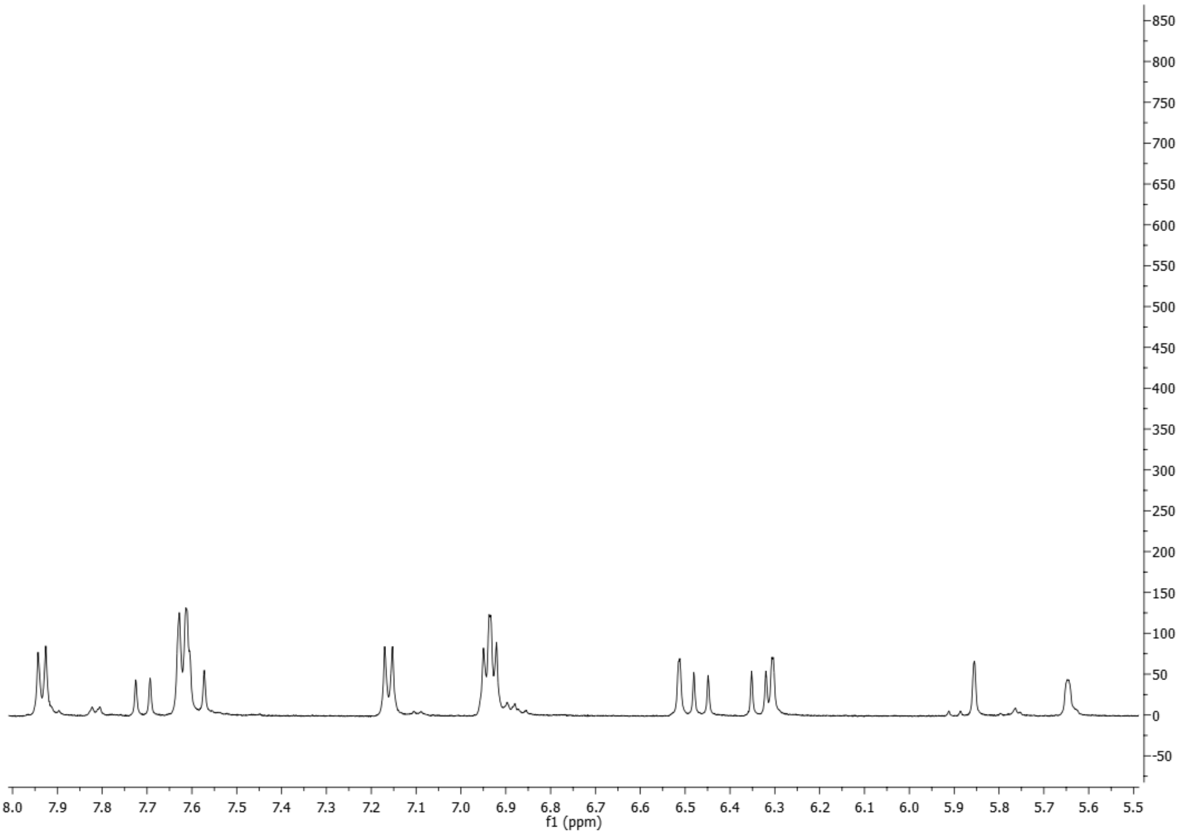


**Figure S6**. $^{1}$H NMR spectrum of kaempferol-3-*O*-α-L-(2”,4”-di-*E*-*p*-coumaroyl)-rhamnoside, **compound 3** (acetone-*d*6, 400 MHz) in the range 8.0 to 5.5 ppm.

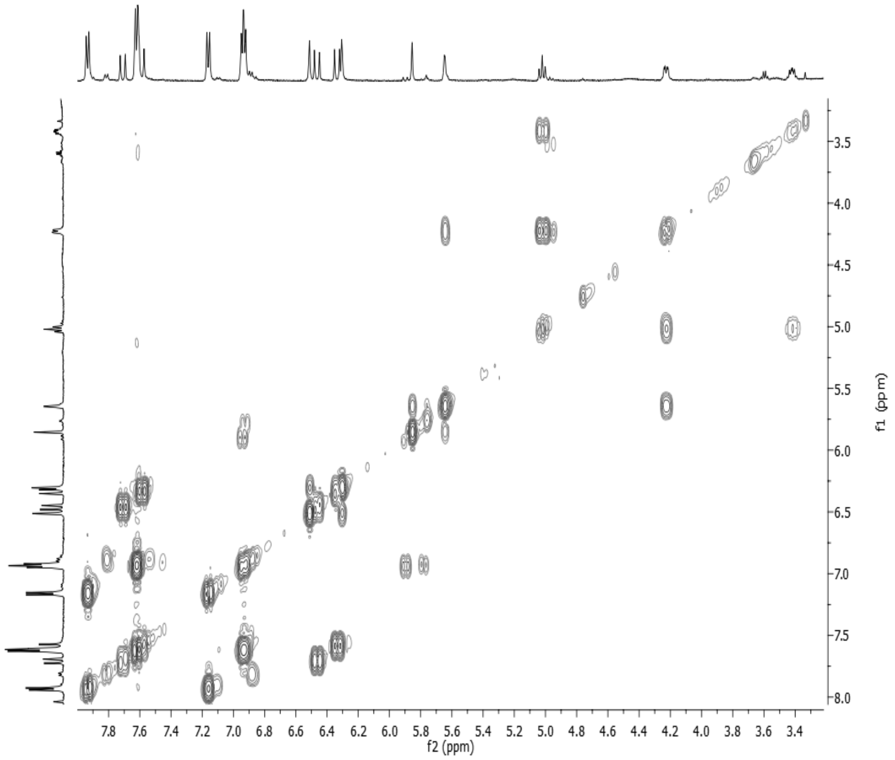


**Figure S7**. COSY spectrum of kaempferol-3-*O*-α-L-(2”,4”-di-*E*-*p*-coumaroyl)-rhamnoside, **compound 3** (acetone-$d_6$, 400 MHz).

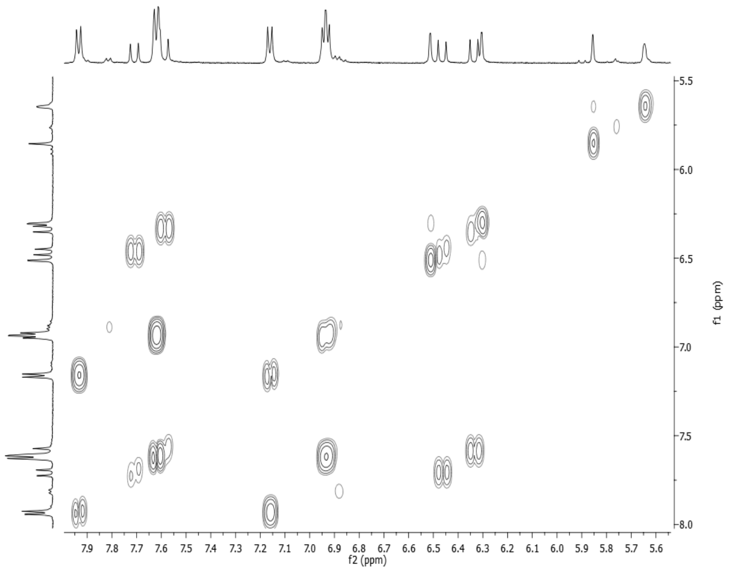


**Figure S8**. COSY spectrum of kaempferol-3-*O*-α-L-(2”,4”-di-*E*-*p*-coumaroyl)-rhamnoside, **compound 3** (acetone-$d_6$, 400 MHz) in the range 8.0 to 5.6 ppm.

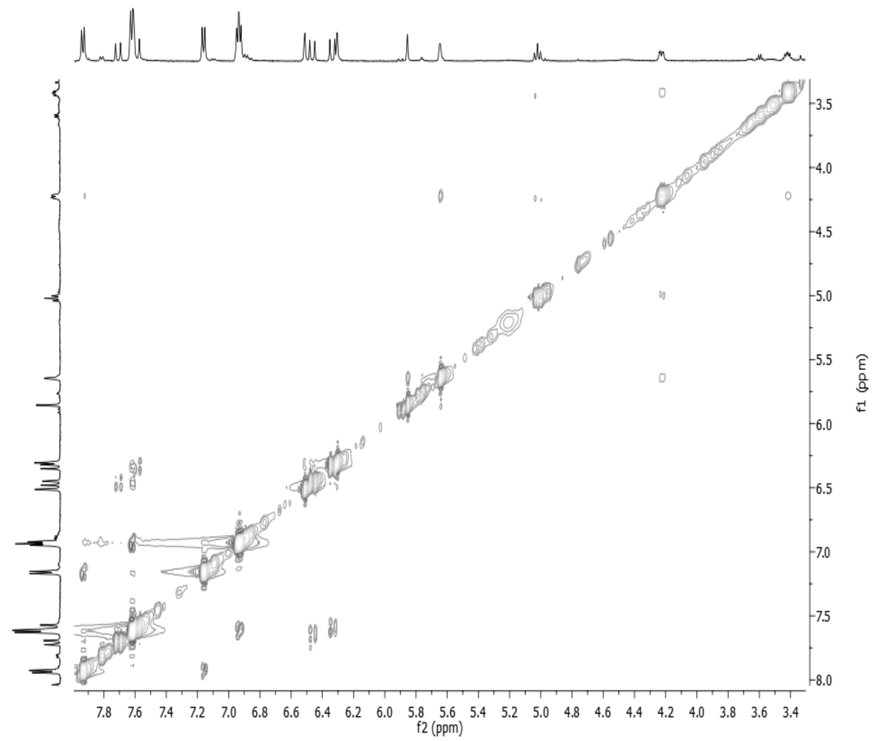


**Figure S9**. NOESY spectrum of kaempferol-3-*O*-α-L-(2”,4”-di-*E*-*p*-coumaroyl)-rhamnoside, **compound 3** (acetone-$d_6$, 400 MHz).

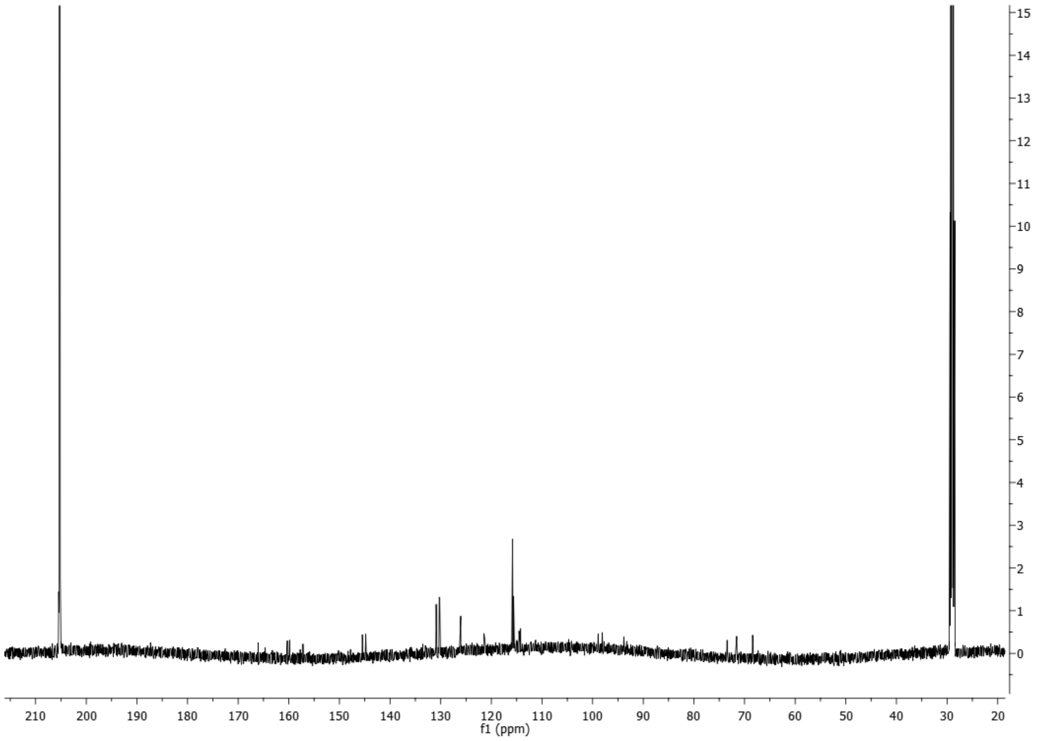


**Figure S10**. $^{13}C$ NMR spectrum of kaempferol-3-*O*-α-L-(2”,4”-di-*E*-*p*-coumaroyl)-rhamnoside, **compound 3** (acetone-$d_6$, 400 MHz).

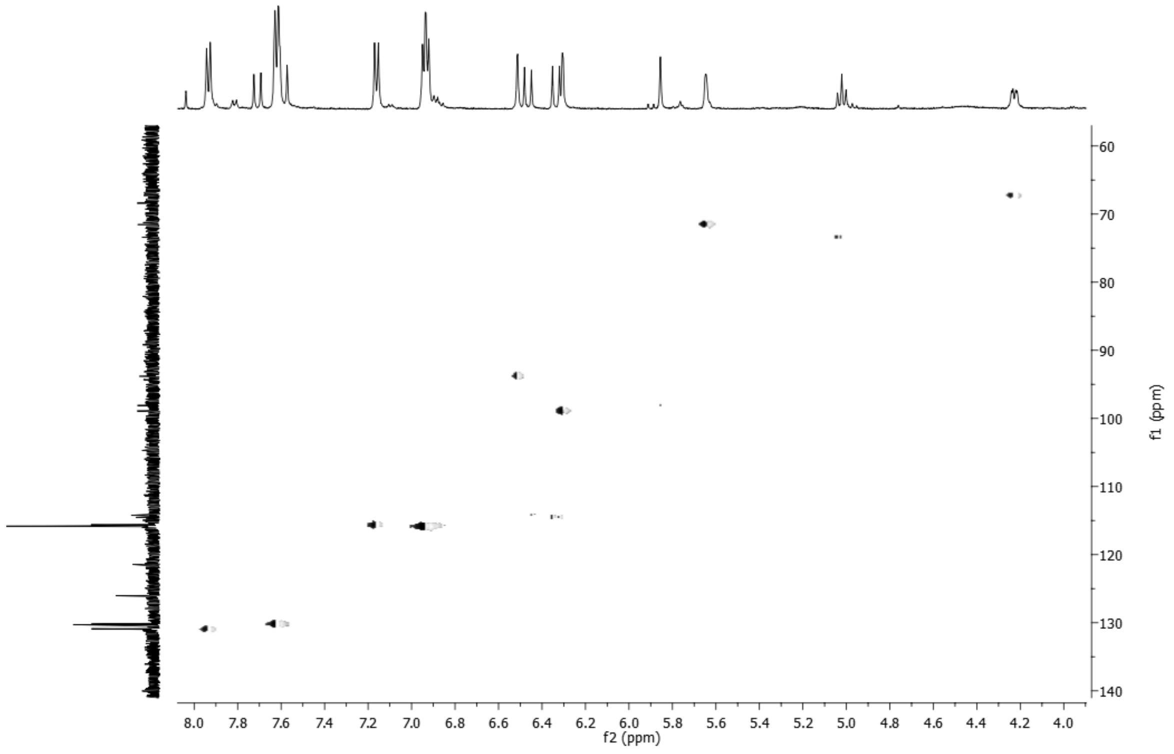


**Figure S11**. HSQC spectrum of kaempferol-3-*O*-α-L-(2”,4”-di-*E*-*p*-coumaroyl)-rhamnoside, **compound 3** (acetone-$d_6$, 400 MHz).

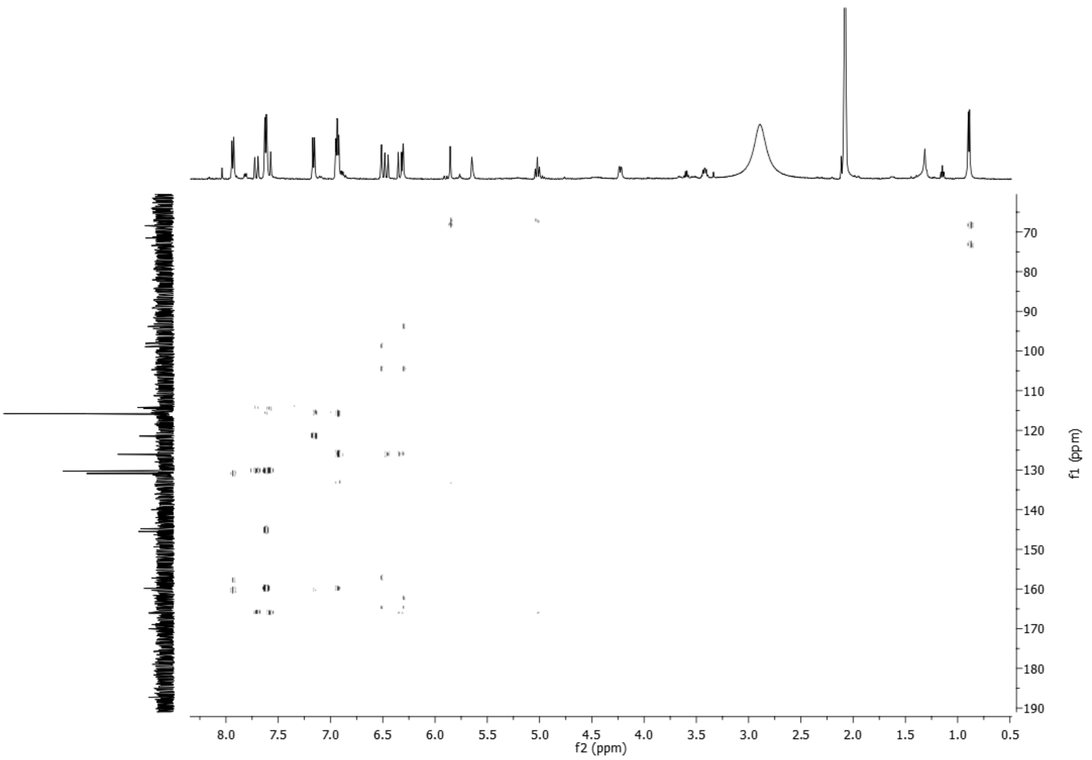


**Figure S12**. HMBC spectrum of kaempferol-3-*O*-α-L-(2”,4”-di-*E*-*p*-coumaroyl)-rhamnoside, **compound 3** (acetone-$d_6$, 400 MHz).

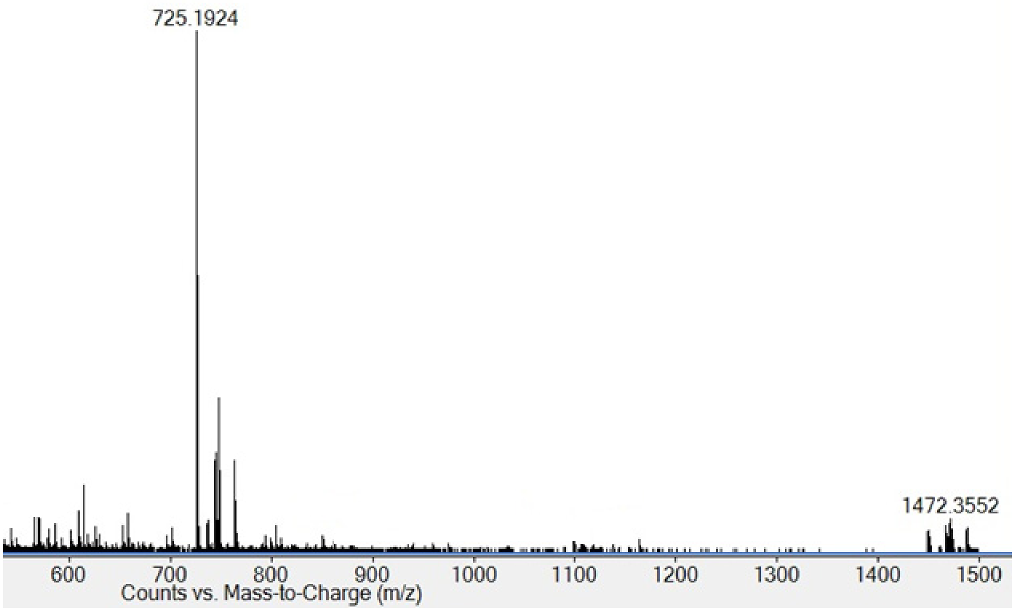


**Figure S13**. ESI MS spectrum of kaempferol-3-*O*-α-L-(2”,4”-di-*E*-*p*-coumaroyl)-rhamnoside, **compound 3** recorded in positive modality.

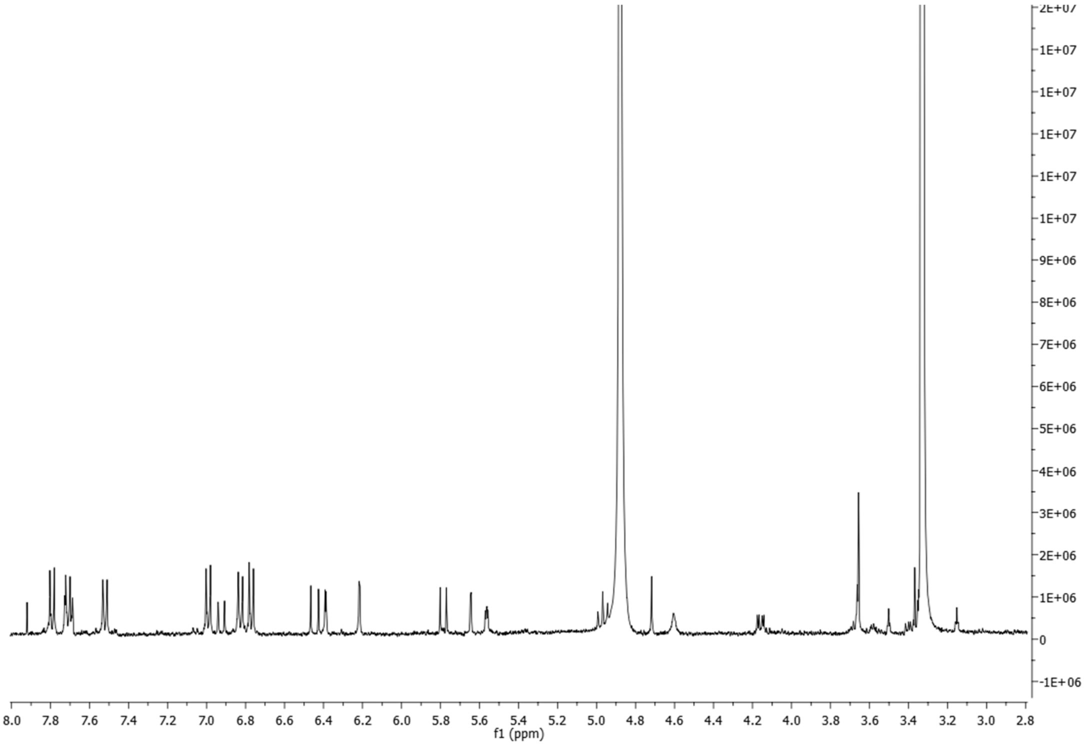


**Figure S14**. $^{1}$H NMR spectrum of kaempferol-3-*O*-α-L-(2”-*E*-*p*-coumaroyl,4”-*Z*-*p*-coumaroyl)-rhamnoside, **compound 4** (MeOD, 400 MHz).

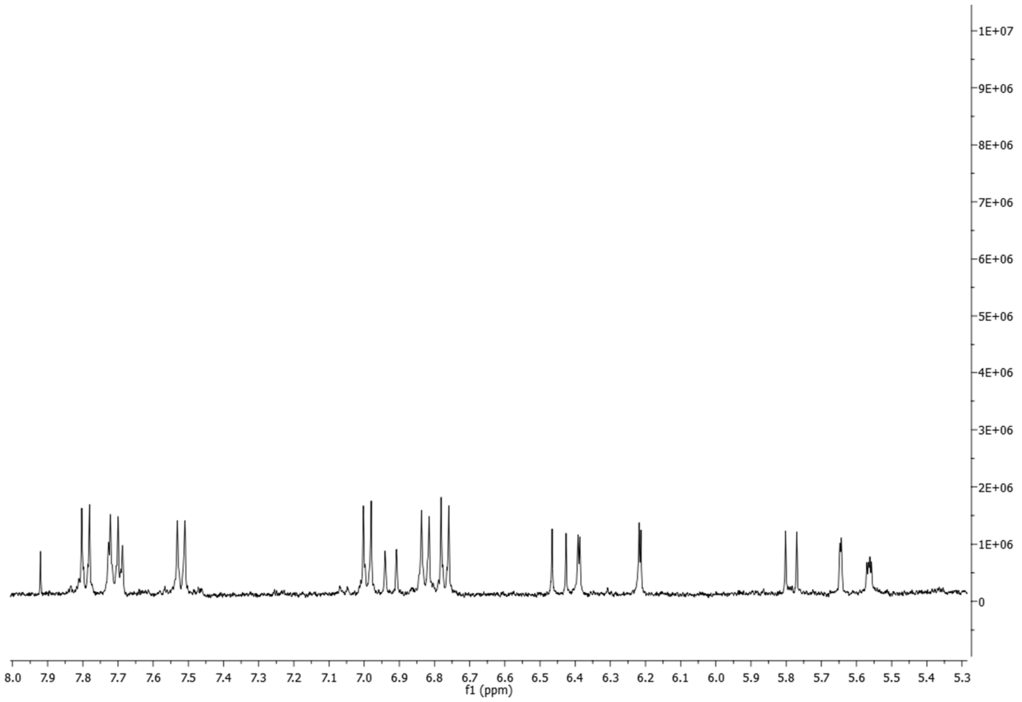


**Figure S15**. $^1$H NMR spectrum of kaempferol-3-*O*-α-L-(2”-*E*-*p*-coumaroyl,4”-*Z*-*p*-coumaroyl)-rhamnoside, **compound 4** (MeOD, 400 MHz) in the range 8.0 to 5.3 ppm.

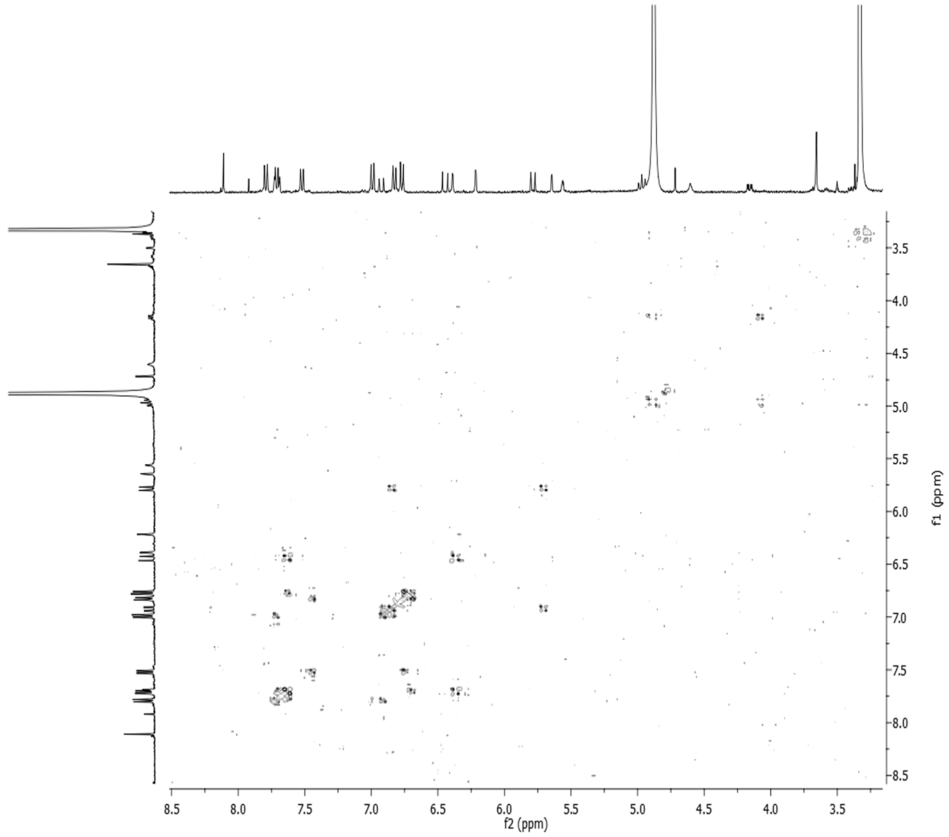


**Figure S16**. COSY spectrum of kaempferol-3-*O*-α-L-(2”-*E*-*p*-coumaroyl,4”-*Z*-*p*-coumaroyl)-rhamnoside, **compound 4** (acetone-$d_6$, 400 MHz).

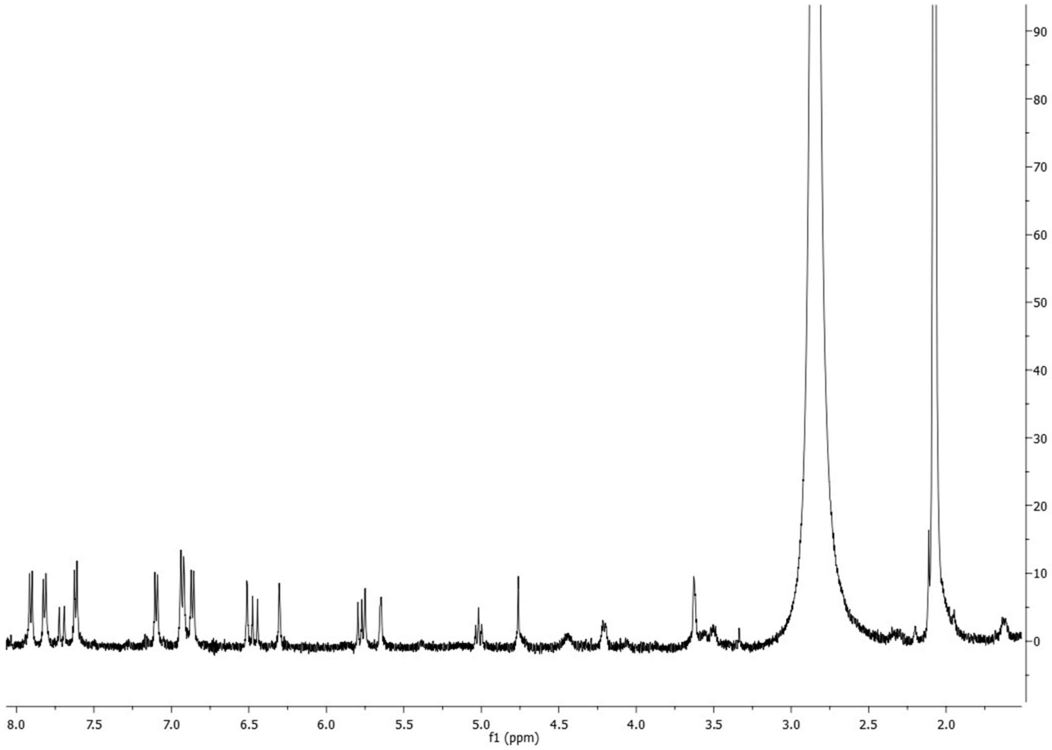


**Figure S17**. ¹H NMR spectrum of kaempferol-3-*O*-α-L-(2”-*E*-*p*-coumaroyl,4”-*Z*-*p*-coumaroyl)-rhamnoside, **compound 4** (acetone-$d_6$, 400 MHz).

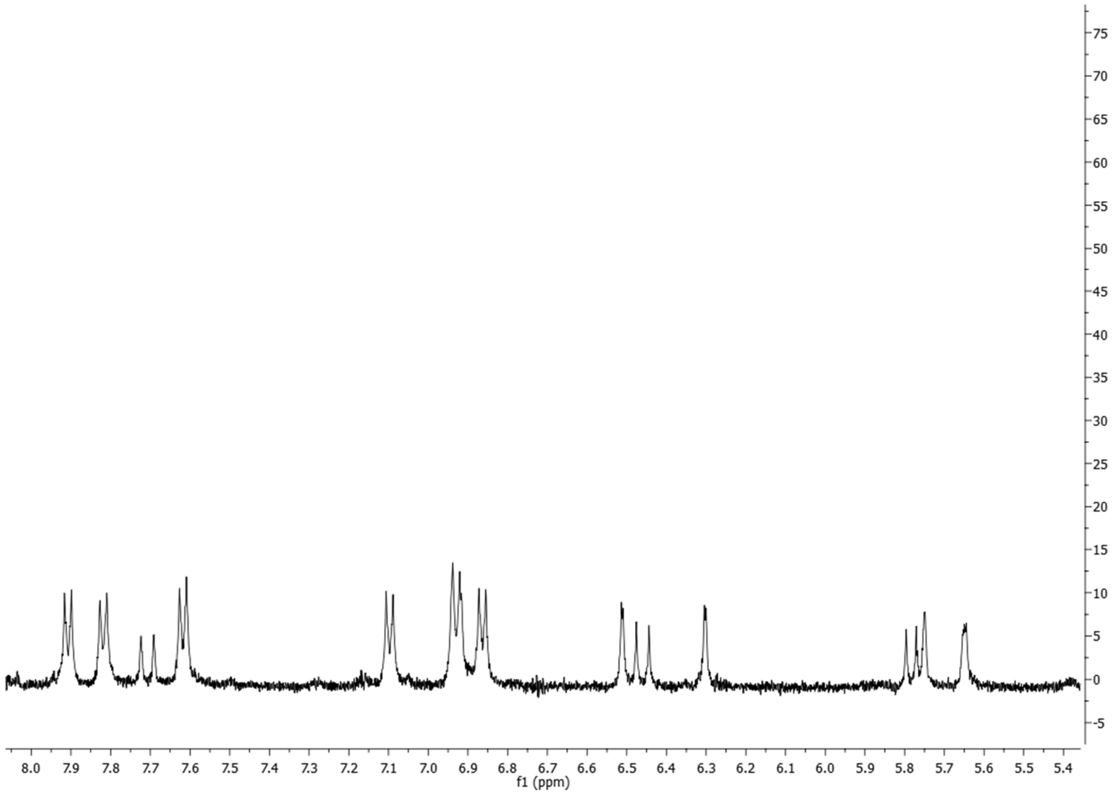


**Figure S18**. ¹H NMR spectrum of kaempferol-3-*O*-α-L-(2”-*E*-*p*-coumaroyl,4”-*Z*-*p*-coumaroyl)-rhamnoside, **compound 4** (acetone-$d_6$, 400 MHz) in the range 8.0 to 5.4 ppm.

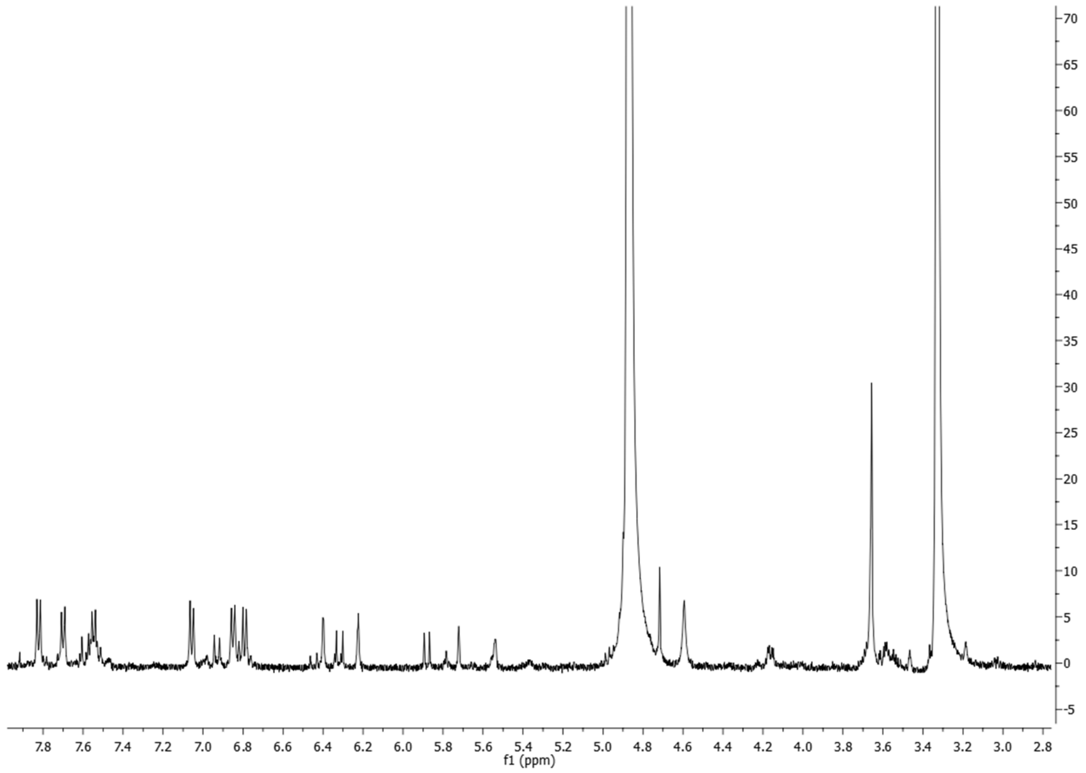


**Figure S19**. $^{1}$H NMR spectrum of kaempferol-3-*O*-α-L-(2”-*Z*-*p*-coumaroyl,4”-*E*-*p*-coumaroyl)-rhamnoside, **compound 5** (MeOD, 400 MHz).

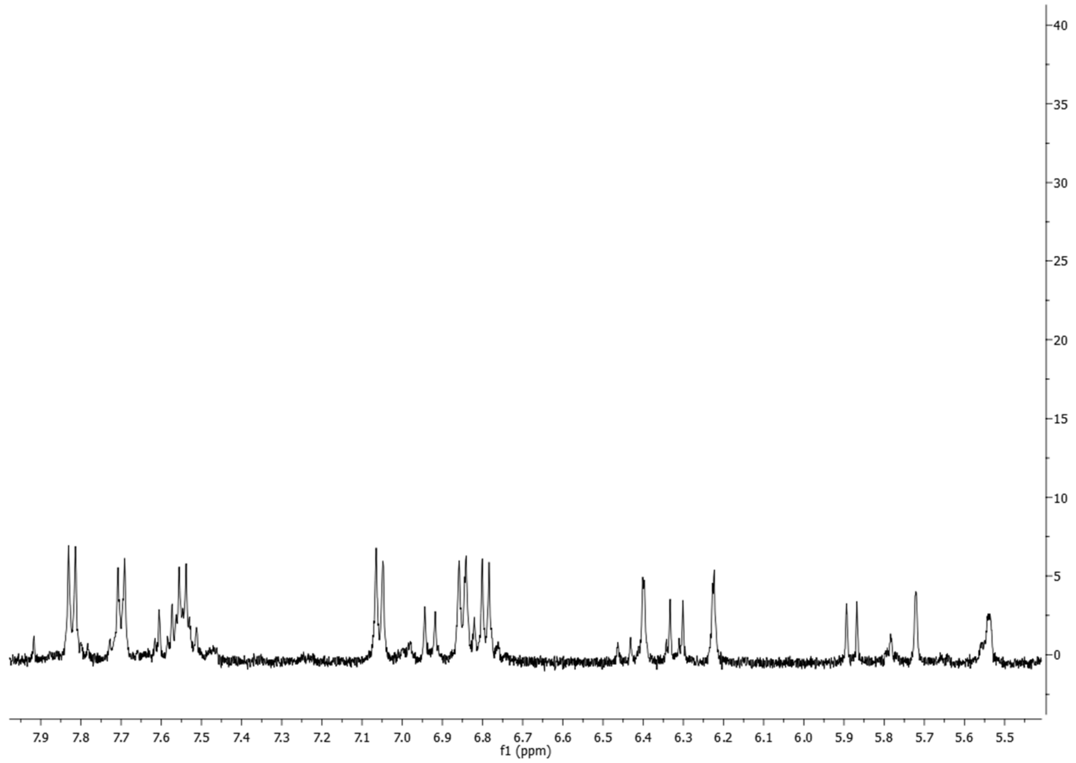


**Figure S20**. $^{1}$H NMR spectrum of kaempferol-3-*O*-α-L-(2”-*Z*-*p*-coumaroyl,4”-*E*-*p*-coumaroyl)-rhamnoside, **compound 5** (MeOD, 400 MHz) in the range 8.0 to 5.4 ppm.

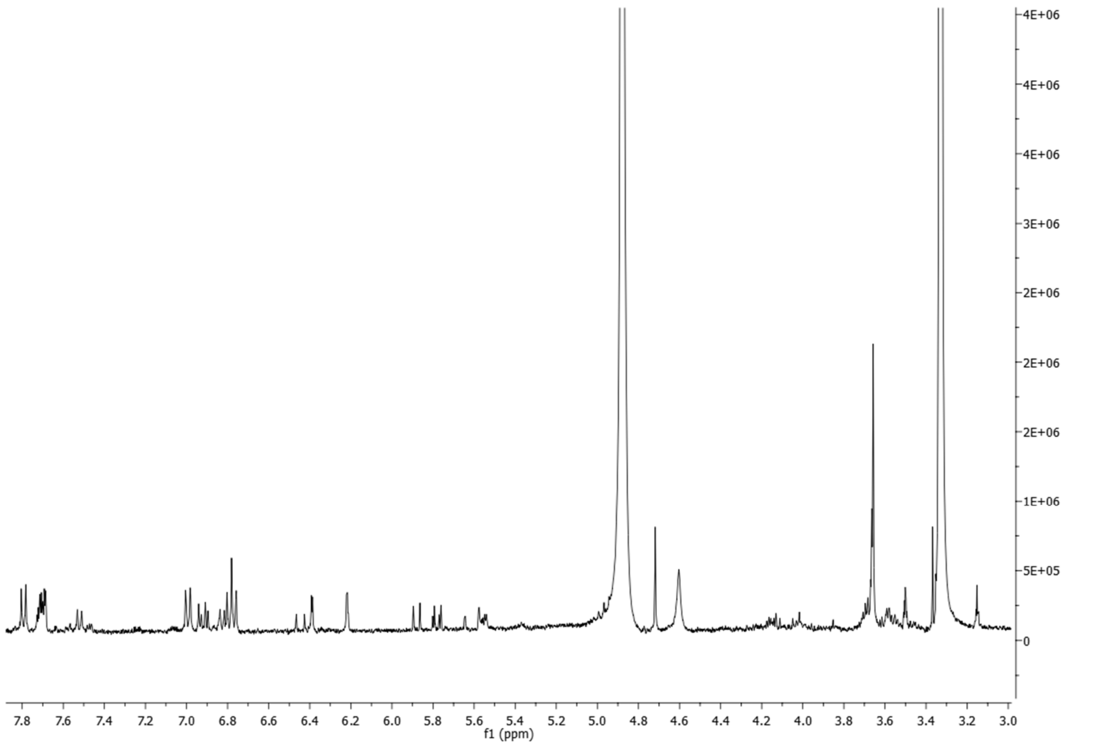


**Figure S21**. $^{1}H$ NMR spectrum of kaempferol-3-*O*-α-L-(2”,4”-di-*Z*-*p*-coumaroyl)-rhamnoside, **compound 6** (MeOD, 400 MHz).

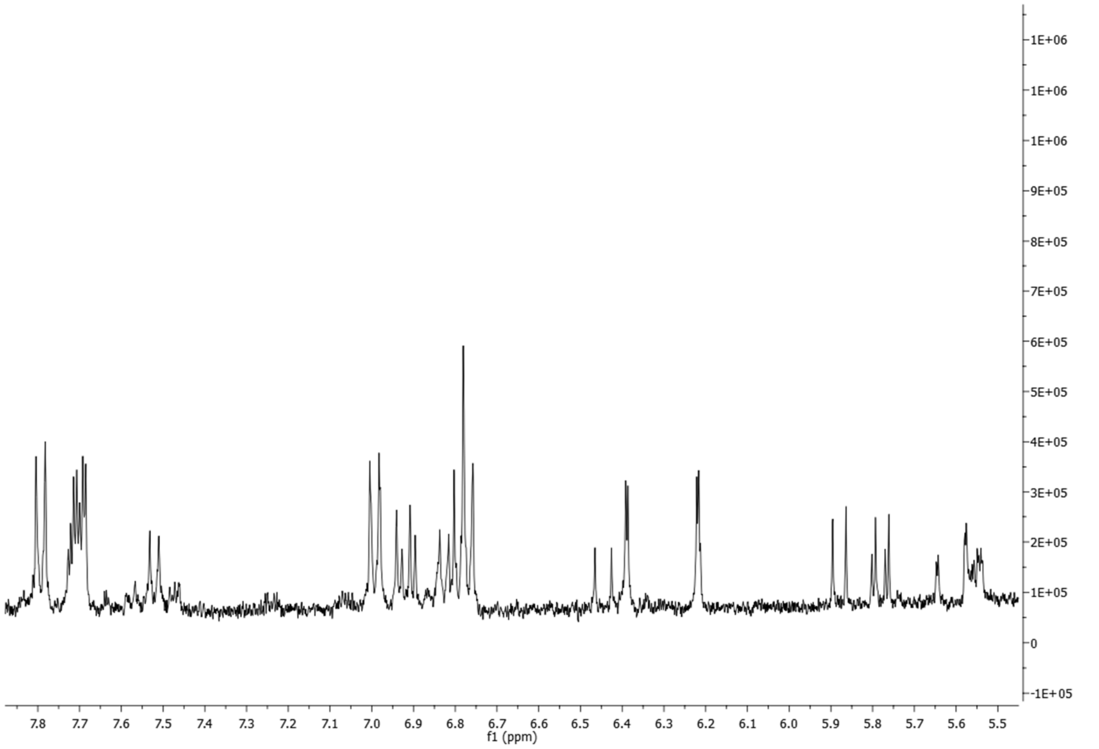


**Figure S22**. $^{1}H$ NMR spectrum of kaempferol-3-*O*-α-L-(2”,4”-di-*Z*-*p*-coumaroyl)-rhamnoside, **compound 6** (MeOD, 400 MHz) in the range 7.9 to 5.5 ppm.

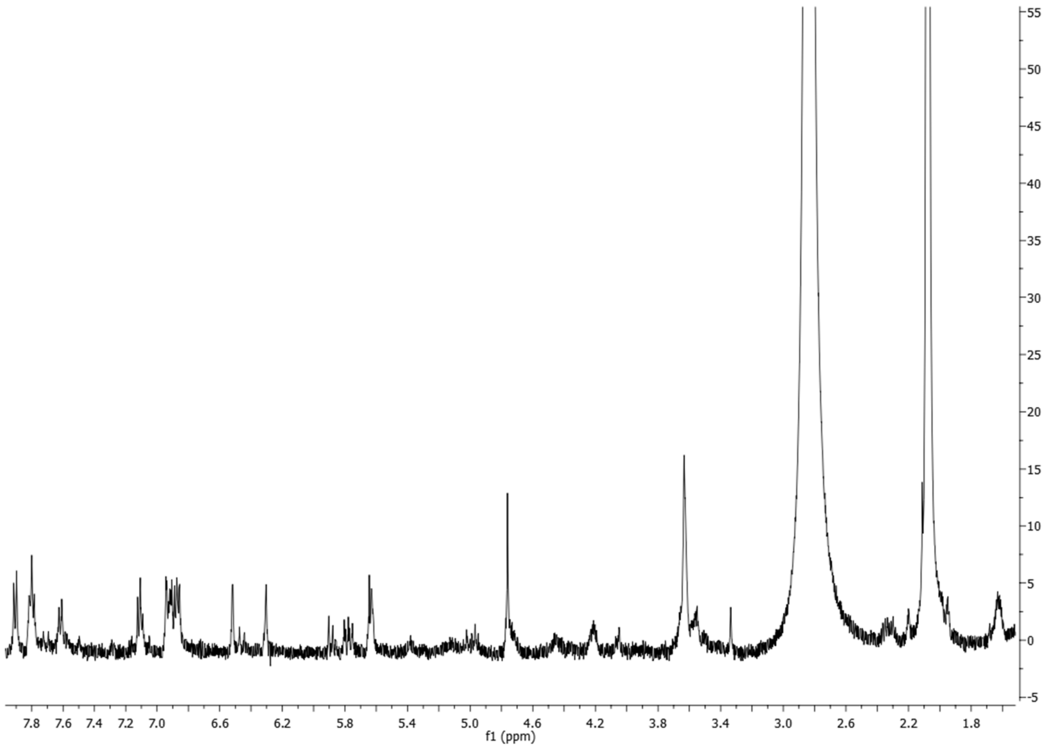


**Figure S23**. $^{1}$H NMR spectrum of kaempferol-3-*O*-α-L-(2”,4”-di-*Z*-*p*-coumaroyl)-rhamnoside, **compound 6** (acetone-$d_6$, 400 MHz).

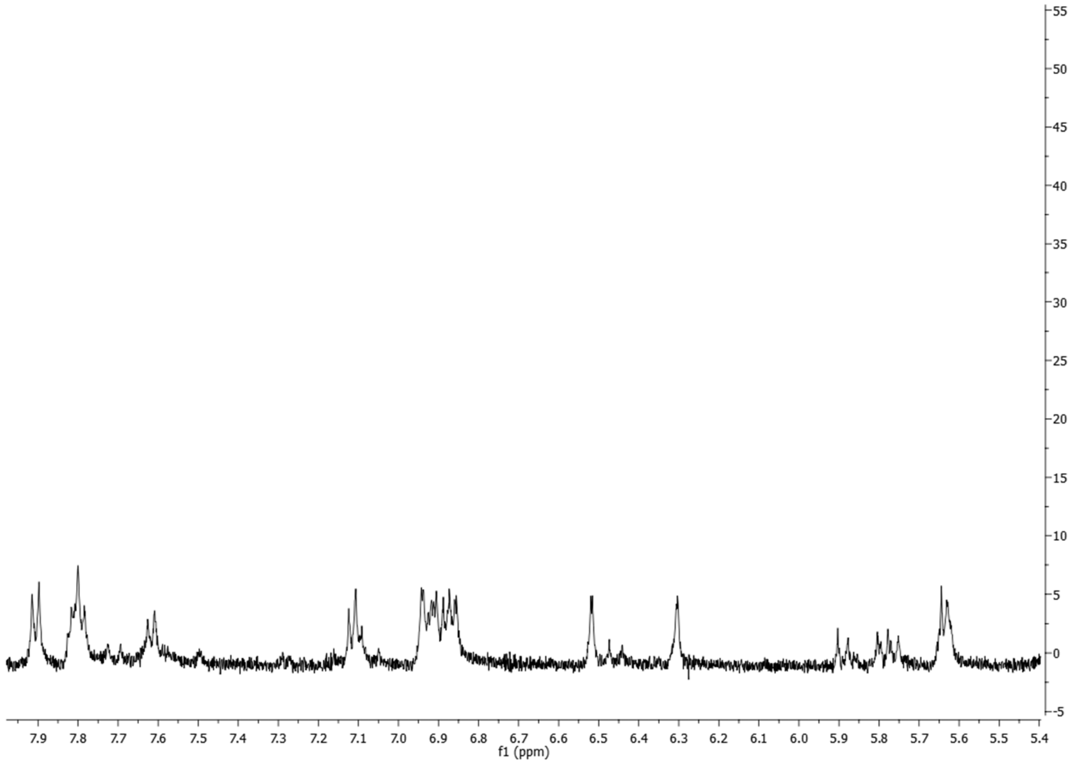


**Figure S24**. $^{1}$H NMR spectrum of kaempferol-3-*O*-α-L-(2”,4”-di-*Z*-*p*-coumaroyl)-rhamnoside, **compound 6** (acetone-$d_6$, 400 MHz) in the range 8.0 to 5.4 ppm.

**Supplementary Table S2**. $MIC_{100}$ values (µg/mL) determined for the essential oils carvacrol and thymol purified from *E. foeminea* hexane extract.

| | $MIC_{100}$ (µg/mL) | |
|---|---|---|
| **Gram-positive strains** | **Carvacrol** | **Thymol** |
| *S. aureus* ATCC 29213 | 100 | 600 |
| *S. aureus* MRSA WKZ-1 | 50 | 2,400 |
| *E. faecalis* ATCC 29212 | 100 | 1,200 |
| **Gram-negative Strains** | | |
| *E. coli* ATCC 25922 | 200 | 600 |
| *S. typhimurium* ATCC 14028 | 100 | 300 |
| *A. baumannii* ATCC 17878 | 100 | 1,200 |

**Supplementary Table S3.** $MIC_{100}$ values (µg/mL) determined for the compounds kaempferol-3-O-(2",4"-di-E-p-coumaryl)-α-L-rhamno-piranoside, kaempferol-3-O-(2"-Z-p-coumaryl,4"-di-E-p-coumaryl)-α-L-rhamno-piranoside, kaempferol-3-O-(2"-E- p-coumaryl,4"-di-Z-p-coumaryl)-α-L-rhamno-piranoside and kaempferol-3-O-(2",4"-di-Z-p-coumaryl)-α-L-rhamno-piranoside purified from *E. foeminea* dichloromethane extract.

| | **$MIC_{100}$ (µg/mL)** | |
|---|---|---|
| | ***S. aureus*** **MRSA WKZ-1** | ***A. baumannii*** **ATCC 17878** |
| **kaempferol-3-*O*-(2",4"-di-*E*-*p*-coumaryl)-*α*-L-rhamno-piranoside** | 0.49 | 1,000 |
| **kaempferol-3-*O*-(2"-*Z*-*p*-coumaryl,4"-di-*E*-*p*-coumaryl)-*α*-L-rhamno-piranoside** | >1,000<br>$MIC_{95}$ = 1,000 | 1,000 |
| **kaempferol-3-*O*-(2"-*E*- *p*-coumaryl,4"-di-*Z*-*p*-coumaryl)-*α*-L-rhamno-piranoside** | >1,000<br>$MIC_{98}$ = 1,000 | 1,000 |
| **kaempferol-3-*O*-(2",4"-di-*Z*-*p*-coumaryl)-*α*-L-rhamno-piranoside** | >1,000<br>$MIC_{98}$ = 1,000 | 1,000 |

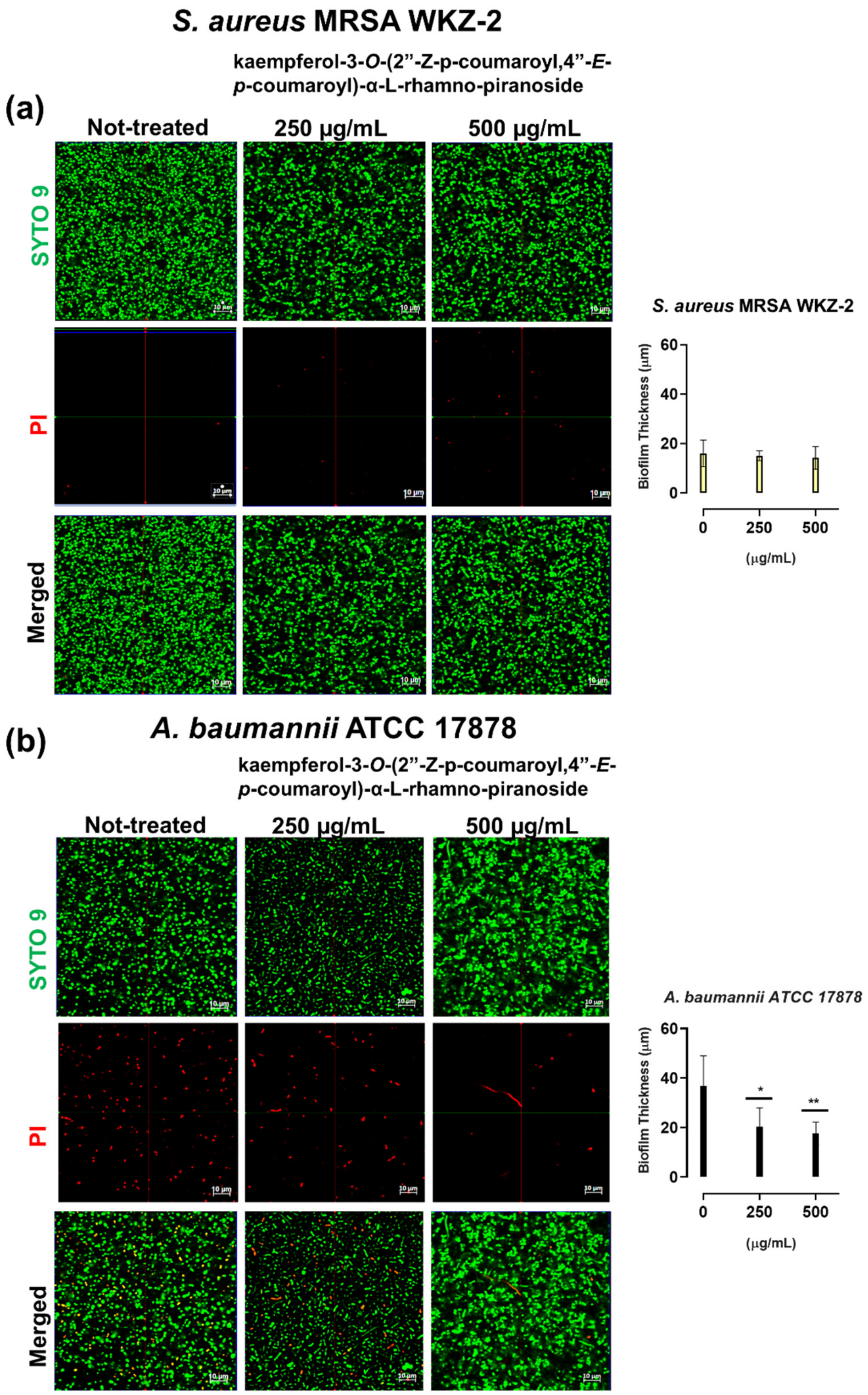


**Figure S25.** Anti-biofilm activity of purified kaempferol-3-*O*-(*2”-Z-p*-coumaroyl*,4”-E-p*-coumaroyl)-α-L-rhamno-piranoside. Effects of kaempferol-3-*O*-(*2”-Z-p*-coumaroyl*,4”-E-p*-coumaroyl)-α-L-rhamno-piranoside have been evaluated on biofilm formation in the case of *S. aureus* MRSA WKZ-2 **(a)**, and *A. baumannii* ATCC 17878 **(b)**. Biofilm cells were stained with LIVE/DEAD BacLight bacterial viability kit (Molecular Probes, Eugene, OR) containing a 1:1 mol/mol ratio of SYTO-9 (green fluorescence, all cells) and propidium iodide (PI; red fluorescence, dead cells). The biofilm images were obtained by confocal z-stack using Zen Lite 2.3 software. All the images were taken under identical conditions. Significant differences were indicated as (*P< 0.05) or (**P< 0.001) for treated *versus* control samples. Each experiment was carried out in triplicate.

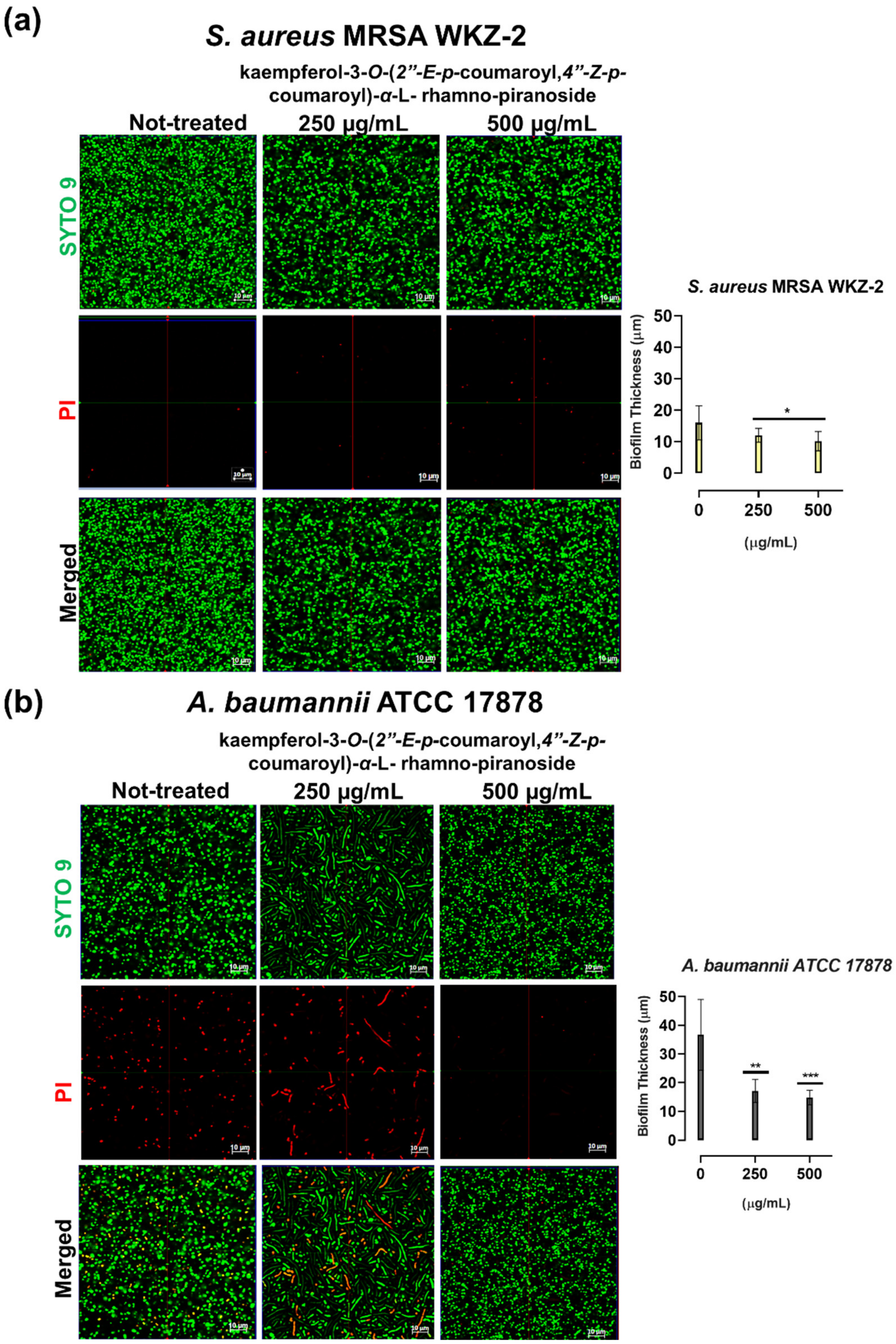


**Figure S26.** Anti-biofilm activity of purified kaempferol-3-*O*-(*2”-E-p*-coumaroyl,*4”-Z-p*-coumaroyl)-*α*-L-rhamno-piranoside. Effects of kaempferol-3-*O*-(*2”-E-p*-coumaroyl,*4”-Z-p*-coumaroyl)-*α*-L-rhamno-piranoside have been evaluated on biofilm formation in the case of *S. aureus* MRSA WKZ-2 **(a)**, and *A. baumannii* ATCC 17878 **(b)** by CLSM. Biofilm cells were stained with LIVE/DEAD BacLight bacterial viability kit (Molecular Probes, Eugene, OR) containing a 1:1 mol/mol ratio of SYTO-9 (green fluorescence, all cells) and propidium iodide (PI; red fluorescence, dead cells). The biofilm images were obtained by confocal z-stack using Zen Lite 2.3 software. All the images were taken under identical conditions. Significant differences were indicated as (*P< 0.05), (**P< 0.001) or (***P< 0.0001) for treated *versus* control samples. Each experiment was carried out in triplicate.

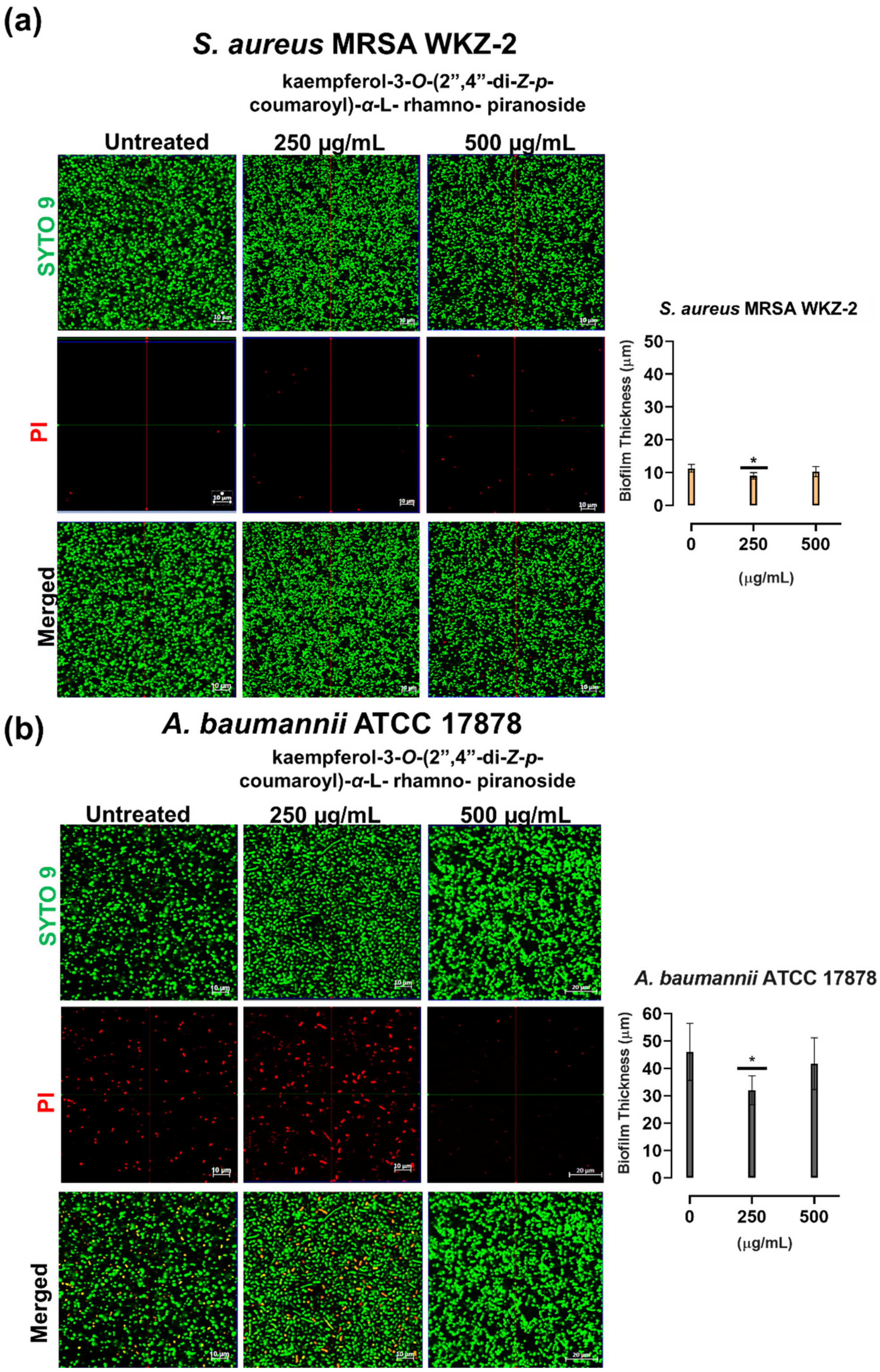


**Figure S27.** Anti-biofilm activity of purified kaempferol-3-*O*-(2”,4”-di-*Z*-*p*-coumaroyl)-*α*-L-rhamno-piranoside. Effects of kaempferol-3-*O*-(2”,4”-di-*Z*-*p*-coumaroyl)-*α*-L-rhamno-piranoside have been evaluated on biofilm formation in the case of *S. aureus* MRSA WKZ-2 **(a)**, and *A. baumannii* ATCC 17878 **(b)** by CLSM. Biofilm cells were stained with LIVE/DEAD BacLight bacterial viability kit (Molecular Probes, Eugene, OR) containing a 1:1 mol/mol ratio of SYTO-9 (green fluorescence, all cells) and propidium iodide (PI; red fluorescence, dead cells). The biofilm images were obtained by confocal z-stack using Zen Lite 2.3 software. All the images were taken under identical conditions. Significant differences were indicated as (*P< 0.05) for treated *versus* control samples. Each experiment was carried out in triplicate.

**Supplementary Table S4.** Details of the intermolecular interactions between the test ligand and *S. aureus* target enzymes tyrosyl tRNA synthetase and sortase A.

| **Interaction between antimicrobial compound and tyrosyl tRNA synthetase** | | | **Interaction between antimicrobial compound and sortase A** | | |
|---|---|---|---|---|---|
| **Interacting amino acid** | **Type of interaction** | **Bond length (Å)** | **Interacting amino acid** | **Type of interaction** | **Bond length (Å)** |
| His 50 | Carbon-hydrogen bond | 3.57 | Val 168 | Hydrogen bond | 2.21 |
| His 47 | Pi-Sigma | 3.92 | Arg 197 | Hydrogen bond | 2.35 |
| His 47 | Pi-Pi T shaped | 5.02 | Ala 92 | Hydrogen bond | 2.66 |
| His 47 | Pi-Pi T shaped | 4.96 | Val 168 | Hydrogen bond | 2.70 |
| Gly 38 | Amide Pi Stacked | 3.52 | Thr 180 | Hydrogen bond | 2.49 |
| Ala 43 | Pi -Alkyl | 5.00 | Val 193 | Pi-Sigma | 3.79 |
| Leu 223 | Pi -Alkyl | 5.37 | Ala 92 | Pi-alkyl | 5.11 |
| | | | Ala 104 | Pi-Alkyl | 5.07 |
| | | | Val 168 | Pi-Alkyl | 4.65 |
| | | | Ile 199 | Pi-Alkyl | 4.54 |
